# Chemical strategies for triggering the immune response to the mycotoxin patulin

**DOI:** 10.1038/s41598-021-02916-6

**Published:** 2021-12-06

**Authors:** Hadyn Duncan, Josep V. Mercader, Consuelo Agulló, Marcos Gil-Sepulcre, Antonio Abad-Somovilla, Antonio Abad-Fuentes

**Affiliations:** 1 Department of Organic Chemistry, Universitat de València, Doctor Moliner 50, 46100 Burjassot, València Spain; 2 Institute of Agrochemistry and Food Technology (IATA), Spanish National Research Council (CSIC), Agustí Escardino 7, 46980 Paterna, València Spain; 3 Present Address: Institute of Chemical Research of Catalonia (ICIQ), Barcelona Institute of Science and Technology (BIST), Av. Països Catalans 16, 43007 Tarragona, Spain

**Keywords:** Immunochemistry, Immunological techniques, Small molecules, Natural hazards, Bioanalytical chemistry, Synthetic chemistry methodology

## Abstract

Mycotoxins represent a major concern for human and animal health because of their harmful effects and high occurrence in food and feed. Rapid immunoanalytical methods greatly contribute to strengthening the safety of our food supply by efficiently monitoring chemical contaminants, so high-affinity and specific antibodies have been generated for almost all internationally regulated mycotoxins. The only exception is patulin, a mycotoxin mainly produced by *Penicillium expansum* for which such a target has not yet been achieved. Accordingly, no point-of-need tests commonly used in food immunodiagnostics are commercially available for patulin. In the present study, three functionalized derivatives conforming to generally accepted rules in hapten design were firstly tested to generate suitable antibodies for the sensitive immunodetection of patulin. However, these conventional bioconjugates were unable to elicit the desired immune response, so an alternative strategy that takes advantage of the high electrophilic reactivity of patulin was explored. Patulin was reacted with 4-bromothiophenol, and the obtained adduct was used to produce antibodies with nanomolar affinity values. These results demonstrated for the first time that targeting the adduct resulting from the reaction of patulin with a thiol-containing compound is a promising approach for developing user-friendly immunoanalytical techniques for this elusive mycotoxin.

## Introduction

The presence of toxic chemicals in food, particularly in products intended for children's consumption, is a matter of great concern to consumers and international health authorities^1^. According to the RASFF annual reports of the European Commission, around 45% of the informative notifications issued in recent years were related to the presence of chemical substances such as pesticides, biotoxins, veterinary drugs, food additives, etc.^2^. Among them, mycotoxins constitute the group of chemical hazards that is responsible for the highest number of notifications, which translates into economic losses for the agri-food industry worth millions of Euros per year. Therefore, strict regulatory guidelines have been established internationally for the major classes of mycotoxins^3^.

**Figure 1:**
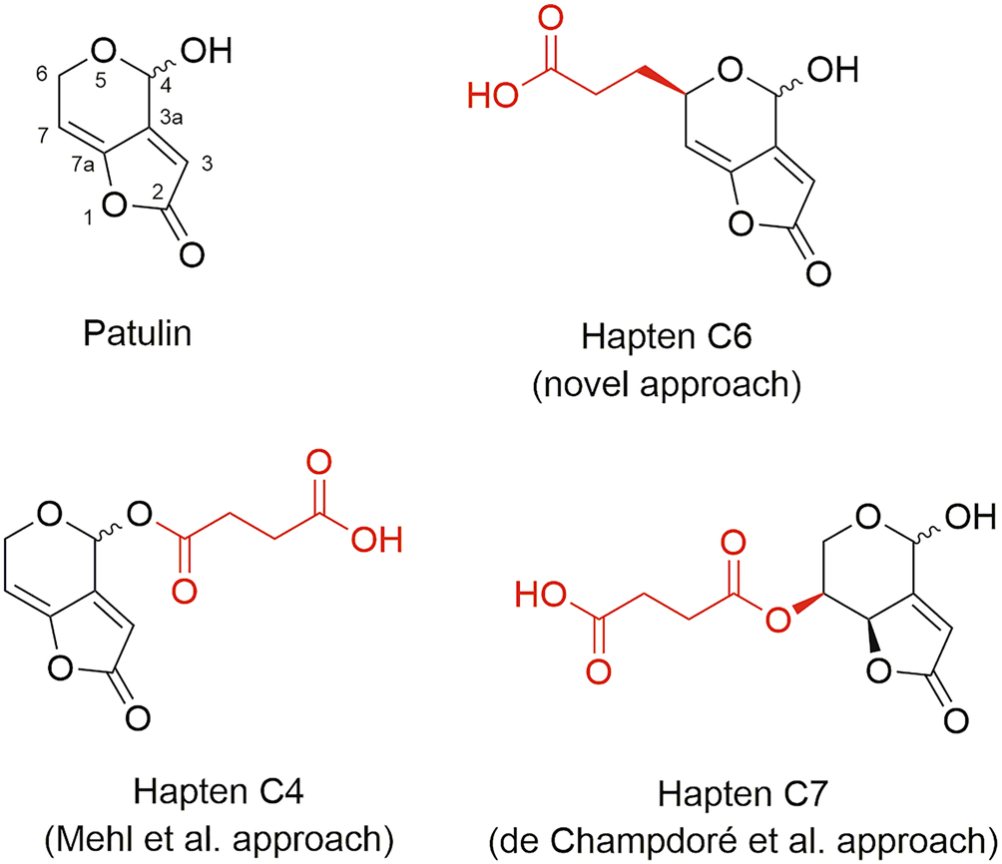
Chemical structures of patulin (showing numbering of carbon atoms) and conventional haptens used in this work for antibody generation.

Patulin [4-hydroxy-4H-furo[3.2-c]pyran-2(6H)-one] (Fig. 1) is a toxic polyketide metabolite mainly produced by *Penicillium expansum*^4^. Different studies in rodents have established that long-term exposure to patulin leads to neurotoxic, immunotoxic, genotoxic, and teratogenic effects. However, the International Agency for Research on Cancer (IARC) has classified patulin in Group 3 as not carcinogenic to humans because of insufficient evidence^5, 6^. The main route of human exposure to patulin is through the ingestion of contaminated fruits, mainly apples and their derived products, like juices and compotes. Patulin is highly stable during food processing, and available technologies aimed at removing or reducing patulin from contaminated juices has been of limited efectiveness so far^7, 8^. The European Union has established maximum allowable levels of patulin in juices (50 µg/kg), apples (25 µg/kg), and in foodstuffs intended for consumption by infants and children (10 µg/kg), and similar criteria were adopted by other countries. International studies on the incidence of patulin in food commodities have shown that a high percentage of the analysed samples contained this mycotoxin^9–11^. More importantly, a series of alerts and product recalls have emerged due to the presence of patulin at levels exceeding the regulatory limits in apple-derived products in the European Union, the United States, Canada, Australia, and Hong Kong, among others^12^.

In the field of mycotoxin monitoring, immunoanalytical techniques, *i.e.,* those based on the use of antibodies as specific recognition elements, are widely implemented in laboratories worldwide, and a high number of immunodiagnostic companies are commercializing rapid tests for the control and detection of regulated mycotoxins, with the remarkable exception of patulin. As a result of the lack of point-of-need methods, the analysis of patulin is mainly carried out by instrumental chromatographic techniques^13, 14^.

The first reported attempt to produce anti-patulin antibodies dates back to 1986^15^. That strategy consisted of introducing a carboxyl linker at the C-4 position of the molecule by reacting patulin with succinic anhydride; however, low antibody titres were obtained and poor recognition of free patulin was observed. This approach was further followed by other authors, with similar disappointing results^16–18^. In 2007, de Champdoré et al*.* reported a different strategy consisting of attaching the spacer arm at the C-7 atom of the patulin framework^19^, but the ability of the so-obtained antibodies to recognize free patulin in solution by standard competitive ELISA was not demonstrated. Accordingly, it remains controversial whether the approaches described to date for the generation of anti-patulin antibodies may result in specific, high-affinity binders suitable for the development of rapid test formats commonly used in food immunodiagnostics, like ELISA and immunochromatographic strips. Moreover, the lack of commercial immunoassays for patulin most likely reflects the inherent difficulties in raising antibodies for this relevant mycotoxin.

The aim of the present study was to generate high-affinity antibodies enabling the sensitive immunodetection of patulin. To this purpose, three functionalized derivatives of the mycotoxin were synthesized and covalently coupled to carrier proteins. All three haptens (Fig. 1) were rationally designed so that a carboxylated aliphatic spacer arm was incorporated at selected positions of the molecular framework of patulin, thus exposing different parts of the molecule to the immune system while keeping its structure essentially intact. The so-called haptens **C4** and **C7** in this work were those previously proposed by the above-mentioned authors, while hapten **C6** is first reported herein. These three haptens constituted what may be called the conventional approach, because the reasoning behind their synthesis was to introduce the linker at selected sites of the molecular framework in order to cause minimum disturbances in the conformation and the electronic distribution of the molecule. Finally, a disruptive novel strategy harnessing the high electrophilic reactivity of patulin was additionally explored in order to trigger the immune response to this elusive mycotoxin.

## Results and discussion

### Hapten synthesis

Patulin monitoring by antibody-based rapid methods is a long-standing goal in food immunodiagnostics, but the small size of the molecule together with its high electrophilic reactivity makes the aim of obtaining suitable antibodies certainly a challenge. Common knowledge in the field of immunoanalytical methods to haptens advices that the functionalized immunizing derivative should closely mimic the conformation, main chemical groups, and electronic distribution of the target molecule^20, 21^. Accordingly, previous attempts have tried to adhere strictly to this general rule. The approach to this problem that was originally proposed by Mehl et al*.* consisted of the incorporation of the spacer arm through the hydroxyl group that is naturally present in patulin via* O*-acylation. This strategy theoretically grants a proper exposure of the mycotoxin to the immune system while causing minimum modifications in the analyte structure^15^. On the other hand, the strategy suggested by de Champdoré et al*.* consisted of introducing the carboxylic linker at position C-7^19^. This derivative displays clear structural differences with respect to patulin. The introduction of the linker at this position is at the expense of suppressing the C7-C7a double bond, and this modification leads to a substantial deviation from the quasi-coplanarity of the patulin framework due to the pyramidalization of both positions—the sp^2^-trigonal hybridization changes to sp^3^-tetrahedral. The authors justified their choice in an attempt to enhance the stability of the derivative by decreasing the reactivity of the molecule to nucleophiles.

**Figure 2:**
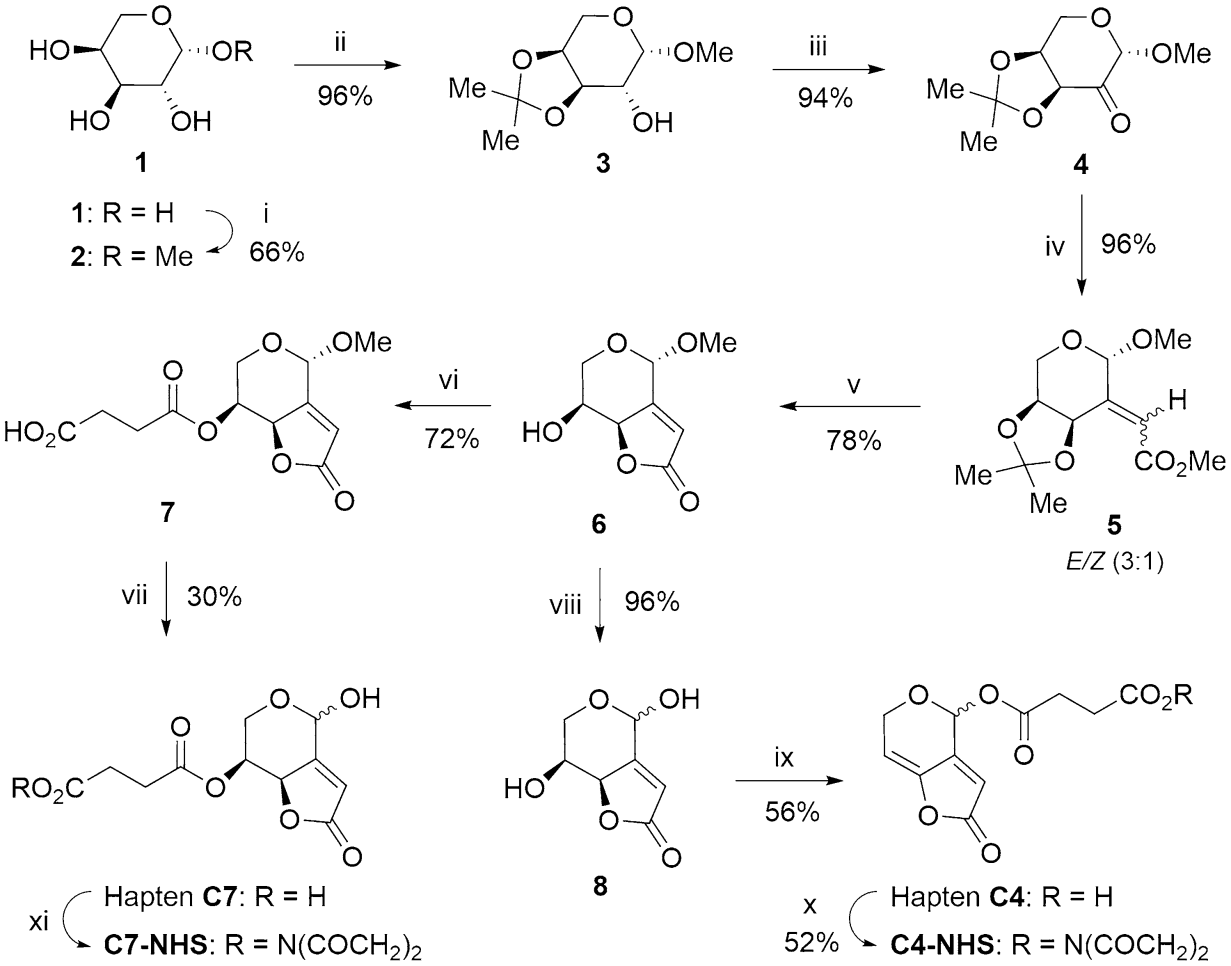
Synthetic route for the preparation of haptens **C4** and **C7**. *Reagents and conditions:* (i) AcCl, MeOH, 50 °C, 4 h; (ii) p-TsOH, Me_2_C(OMe)_2_; acetone, rt, 22 h; (iii) (COCl)_2_, DMSO, CH_2_Cl_2_, − 78 °C, 30 min, then Et_3_N, − 78 °C to rt, 1 h ; (iv) Ph_3_P = CHCO_2_Me, benzene, 50 °C, 6 h; (v) HCl 1.2 M, MeOH, reflux, 2.5 h; (vi) (CH_2_CO)_2_O, DMAP, THF, rt, 16 h; (vii) TFA/H_2_O (9:1), 45 °C, 16 h; (viii) TFA/H_2_O (9:1), 50 °C, 16 h; (ix) (CH_2_CO)_2_O, EtN_3_, DMAP, THF, rt, 1 h; (x) EDC·HCl, NHS, acetone, rt, 24 h; (xi) DCC, NHS, DMF, rt, 24 h (see experimental details in the SI file).

The two above-mentioned approaches are herein represented by haptens **C4** and **C7**, which were synthesized as summarized in Fig. 2. (*S*)-patulin methyl ether (**6**) was obtained with an overall yield of 44.6% starting from L-(+)-arabinose, essentially as previously reported^22^. From this derivative, hapten **C4** was obtained in two straightforward steps with a yield of *ca*. 54%, whereas hapten **C7** was attained with a yield of *ca*. 22%, also in two steps.

**Figure 3:**
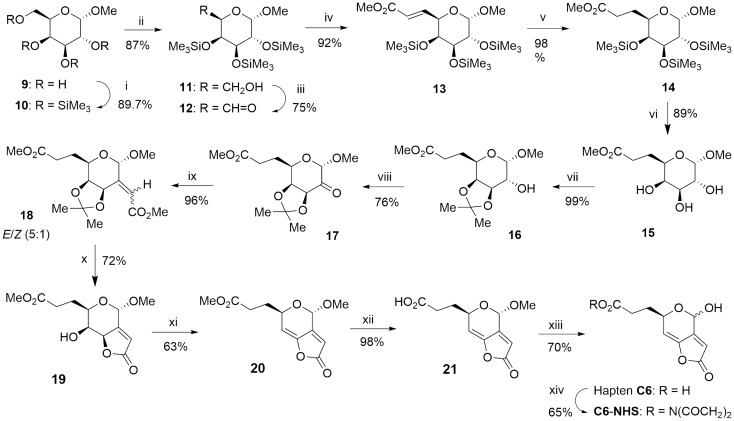
Synthetic route for the preparation of hapten **C6**. *Reagents and conditions:* (i) HMDS, TMSOTf, CH_2_Cl_2_, rt, 2 h; (ii) NH_4_OAc, MeOH/CH_2_Cl_2_ (1:1), rt, 25 h; (iii) TPAP, NMO, 4 Å MS, CH_2_Cl_2_, 1.50 h; (iv) Ph_3_P = CHCO_2_Me, benzene, rt, 6 h; (v) RhCl(PPh_3_)_3_, H_2_, 4 atm, THF, rt, 24 h; (vi) DOWEX 50 W X8, MeOH, rt, 18 h; (vii) p-TsOH, Me_2_C(OMe)_2_, acetone, rt, 60 h; (viii) TPAP, NMO, 4 Å MS, CH_2_Cl_2_, 2.50 h; (ix) Ph_3_P=CHCO_2_Me, benzene, 50 °C, 6 h; (x) MeOH, 1.2 M HCl, reflux, 2.5 h; (xi) MsCl, pyridine, 0 °C to rt, 8 h; (xii) Lipase acrylic resin, PB/THF (4:1), rt, 24 h; (xiii) TFA/H_2_O (9:1), 50 °C, 4 h; (xiv) EDC∙HCl, NHS, MeCN, rt, 3 h (see experimental details in the SI file).

With the aim of attaining the lowest possible structural modification of patulin, an alternative approach was envisioned (hapten **C6** in Fig. 3). Patulin and hapten **C6** share the same skeleton and functional groups. Additionally, the introduction of the hydrocarbon chain through the position C-6 implies a notable increase in steric hindrance near the adjacent unsaturated position (C-7), thus resulting in a reduced susceptibility of C-7 to nucleophilic addition, which is deemed the main cause of the remarkable reactivity of patulin.

The synthetic route for the preparation of this hapten started from commercially available methyl α-D-galactopyranoside (**9**) (Fig. 3). After protecting all hydroxyl groups as trimethylsilyl ethers (**10**), the primary hydroxyl group was selectively desilylated (**11**) and converted to the corresponding aldehyde (**12**) by oxidation with tetrapropylammonium perruthenate (TPAP) and *N*-methylmorpholine-*N*-oxide (NMO). The completion of the carboxylated spacer arm (as methyl ester) first involved Wittig reaction with the stabilized ylide (carbomethoxymethylene)triphenylphosphorane, followed by hydrogenation of the generated double bond under homogeneous conditions using Wilkinson’s catalyst. Once the insertion of the linker was accomplished (**14**), the preparation of the common hapten core was carried out starting with the deprotection of the hydroxyl groups under mild conditions (**15**). The hydroxyl groups at the C-4 and C-5 positions were then selectively re-protected by the formation of an isopropylidene ketal (**16**), thus allowing the further oxidation of the remaining hydroxyl group with TPAP-NMO. Wittig olefination of the obtained ketone (**17**), using the same ylide as above, afforded a 5:1 mixture of *E*/*Z* stereoisomers (**18**) that could not be separated by column chromatography, but that after the hydrolysis of the isopropylidene group under acid conditions led directly to the unsaturated γ-lactone **19** in 69% global yield from **17**. Transformation of the axially disposed hydroxyl group at the C-7 position into a mesylate leaving group allowed for the formation of the C7‒C7a double bond via a bimolecular β-elimination reaction (E2), thus completing the functionalization of the hapten framework characteristic of patulin (**20**). Finally, hydrolysis of the methyl ester group under mild conditions, using a lipase from *Candida antarctica* immobilized on an acrylic resin, followed by acid-catalysed hydrolysis of the acetal methoxy group of **21**, afforded the desired hapten **C6** as a 2:1 mixture of epimers at the hemiacetal carbon. The overall yield of the 13-steps sequence leading to the hapten **C6** was about 11%.

### Hapten activation and preparation of bioconjugates

As shown in the previous figures, all three haptens were equipped with a free carboxylic acid at the end of the linker, so they could be readily activated through carbodiimide-mediated esterification with *N*-hydroxysuccinimide (NHS). The thus obtained *N*-hydroxysuccinimidyl esters of haptens **C4** and **C6**, **C4**–NHS and **C6**–NHS, respectively, were isolated and purified prior to their conjugation to the carrier proteins in order to prevent undesired secondary reactions and to achieve a fine control over coupling conditions. The structure of both active esters (obtained in 52% and 65% isolated yield, respectively) was confirmed by ^1^H NMR spectroscopy. In the case of hapten **C7** we observed a significant loss of the linker during the activation step that led to a mixture of the corresponding active ester (**C7**-NHS) and patulin—up to 30%, which increased notably when trying to separate it from the active ester by column chromatography. The easy loss of the spacer arm in hapten **C7**—a bimolecular E2 elimination process—can be attributed to the anti-coplanar relationship that exists between the (3-carboxypropanoyl)oxy moiety at C-7 (a relatively good leaving group) and the hydrogen atom at the C-7a allylic position. Given the difficulties encountered for the isolation and chromatographic purification of **C7**–NHS, it was used without further purification for the preparation of bioconjugates. It must be noted that the instability of the linker is a matter of concern when dealing with hapten activation and conjugation because it may occur later in vivo following inoculation with the bioconjugate.

**Figure 4:**
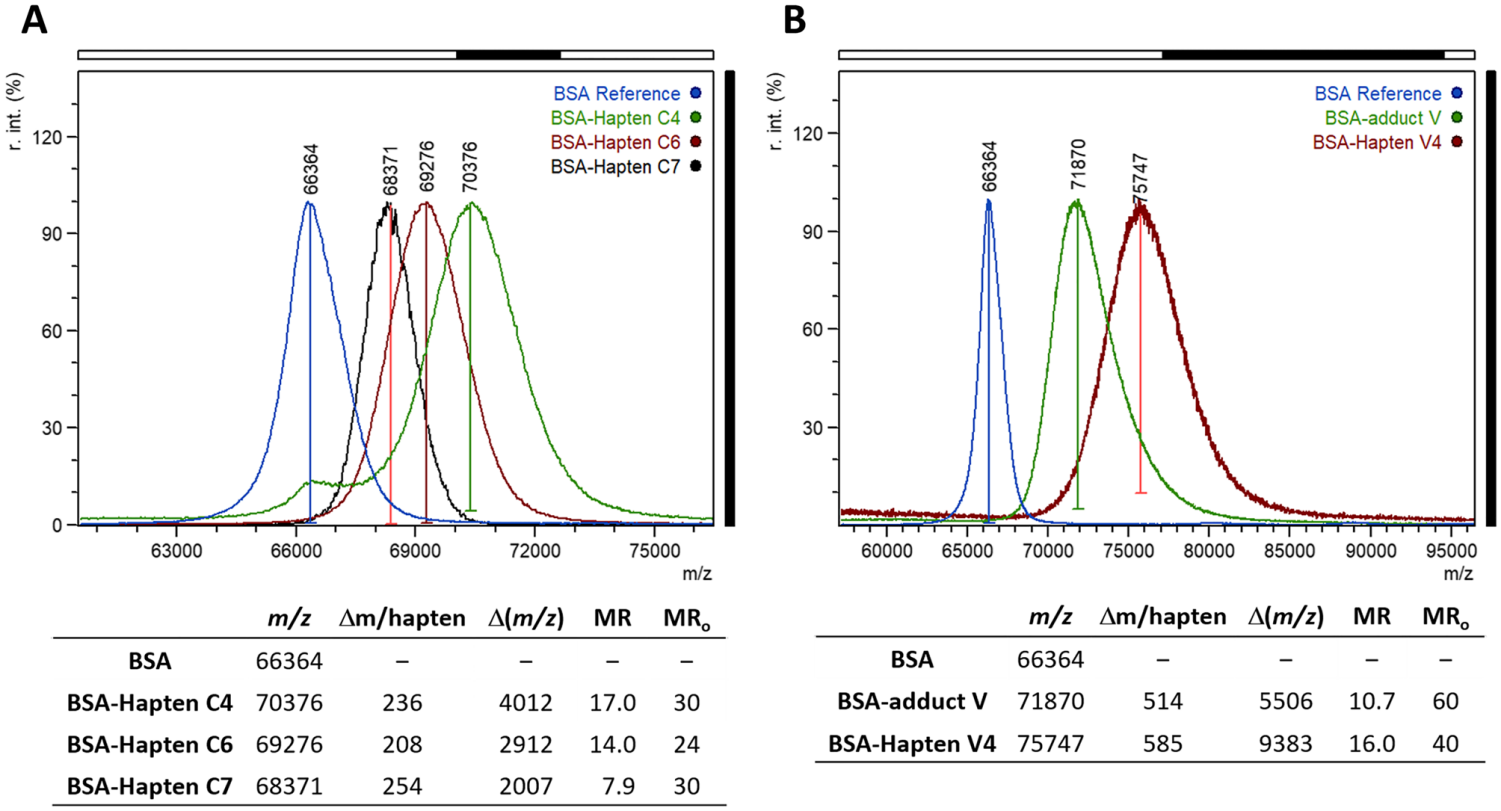
(**A**) MALDI-TOF mass spectra (singly charged ions) of BSA (blue) and the corresponding conjugates with haptens **C4** (green), **C6** (red), and **C7** (black). (**B**) MALDI-TOF mass spectra (singly charged ions) of BSA (blue) and the corresponding conjugates with adduct **V** (green) and hapten **V4** (red). Spectrum intensities are normalized. MR_o_ and MR are the initial and final hapten-to-protein molar ratios, respectively.

Bovine serum albumin (BSA) and ovalbumin (OVA) were chosen as carrier proteins. The activated haptens were added to BSA and OVA at 30-fold and 10-fold molar excess, respectively. The bioconjugates were dialysed after size-exclusion chromatography purification, and the final hapten densities were measured by MALDI-TOF analysis. In the case of BSA (Fig. 4A), the found molar ratios were 17.0, 14.0, and 7.9 for haptens **C4**, **C6**, and **C7**, respectively, whereas the OVA bioconjugates had values ranging from 2 to 7. It is worth noting that the coupling yields for hapten **C7** were substantially lower, which is likely due to the aforementioned ease with which the elimination of the spacer arm occurs in this hapten.

### Evaluation of the immune response

The polyclonal antibodies derived from rabbits immunized with BSA–hapten conjugates were assayed by indirect competitive ELISA. Low-to-moderate titres were observed, and none of the six antibodies showed significant recognition towards free patulin in solution, even at a concentration of 10 µM. When de Champdoré et al*.* addressed the production of antibodies to patulin, they introduced the linker at the C-7 position of the molecule because of the high electrophilic character of the mycotoxin. The authors wisely reasoned that the reactivity of the molecule would be seriously compromised if the double bond in the dihydropyran ring was removed. However, attending to the outstanding previous works by Fliege and Metlzer, who first reported a comprehensive study on the strong reactivity of patulin towards nucleophiles^23, 24^, this scenario seems to be only partially true. Thus, when patulin reacts with a thiolated compound, an addition to the C-7 position does happen first, but the reaction does not stop at this point. On the contrary, the resulting derivative is still able to react with additional nucleophiles giving rise to a high number of different compounds. Therefore, the studies by Fliege and Metlzer provide a feasible explanation of why haptens **C4**, **C6**, and **C7** were not able to elicit the production of anti-patulin antibodies with desirable binding features. Even if the NHS esters of the haptens are purified and characterized before protein coupling, and conjugates are unequivocally identified by MALDI-TOF spectrometry, as it was done in this study, haptens grafted in the conjugate may react in vivo with biological *S-*nucleophiles (and even *N*-nucleophiles) following inoculation of experimental animals^25, 26^. These reactions would lead to a severe modification of the molecular structure of the hapten, and therefore antibodies unable to recognize the intact mycotoxin would be generated.

Despite no commercial rapid tests for patulin immunodetection are available, several authors have reported the use of a rabbit polyclonal antibody from Agrisera to develop rather complex immunoanalytical strategies for the determination of patulin^27–29^. Encouraged by those studies, this antibody—kindly supplied by the company − was tested using our collection of bioconjugates. Antigens OVA–**C4** and OVA–**C7** were recognized by this antiserum, although with low titres. However, no inhibition by patulin in solution was either detected in competitive assays. To gain further insights into the binding properties of this antibody, OVA was modified with succinic anhydride, the spacer arm used in haptens **C4** and **C7**. This pseudo-conjugate, with no patulin attached, was also able to interact with the antibody, indicating that it may actually be recognizing the spacer arm that links the hapten to the carrier. These results suggest that haptens **C4** and **C7** could have partially lost the patulin skeleton during conjugation and/or immunization, leaving exclusively the succinyl chain bound to the protein. This possibility is supported by our observations during the synthesis and activation of hapten **C7** and previous works on the reaction of acetyl patulin (structurally and functionally related to hapten **C4**) with nucleophiles^22, 23^.

Overall, these experiments strongly support the idea that the generation of anti-patulin antibodies through conventional approaches aimed at preserving the structural integrity of the mycotoxin is not probably feasible. The high reactivity of the mycotoxin to nucleophiles, combined with the small size of the molecule and the instability of the hapten, either in vitro or in vivo, are factors likely underpinning this conclusion. In fact, the toxicity of the mycotoxin relies on its capacity to modify cysteine residues in proteins/enzymes, thus inactivating or reducing their activity and affecting the function of essential cellular systems, directly or indirectly^30, 31^. Such conclusion not only applies to haptens **C4** and **C7** with a succinyl spacer arm, but also to hapten **C6**, a derivative that was synthesized through an elaborated synthetic pathway and that does not raise doubts about its stability during purification and conjugation.

### Exploring an innovative approach

**Figure 5:**
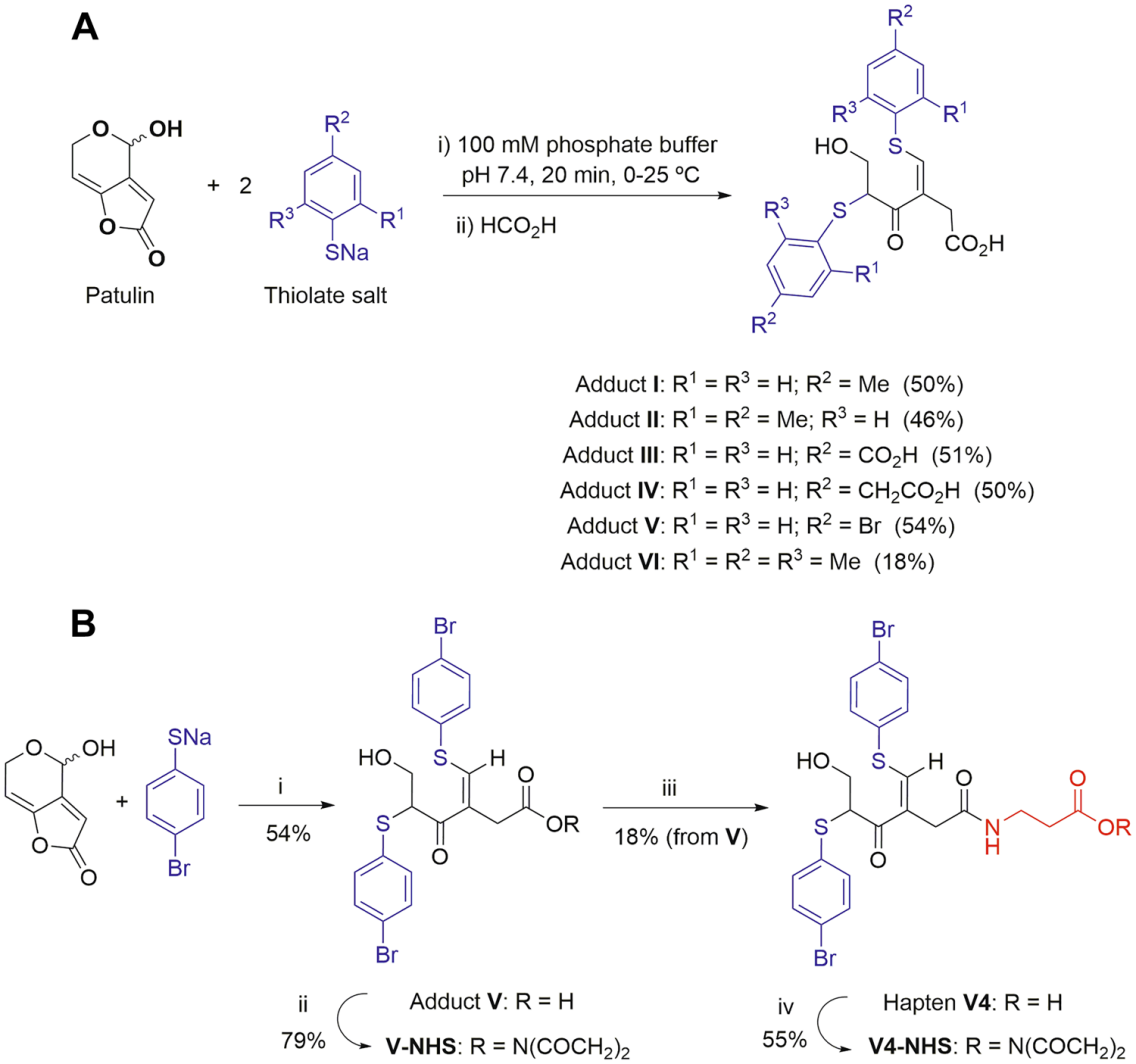
(**A**) Reaction of patulin with the studied aromatic thiols and structures of the major adducts that are formed. The coloured fragments correspond to the thiolate framework incorporated into the adduct structure. The yields (in parentheses) were calculated after chromatographic purification in order to characterize the adducts by spectroscopic methods; however, with no workup the reactions are nearly quantitative. (**B**) Synthesis and activation of adduct **V** and hapten **V4**. *Reagents and conditions:* (i) 100 mM sodium phosphate buffer, pH 7.4, 0 °C, 20 min; (ii) EDC·HCl, NHS, CH_2_Cl_2_, − 20 °C, 1 h; (iii) β-alanine, MeCN/H_2_O (1:1), from 0 °C to rt, 2 h; (iv) EDC·HCl, NHS, CH_2_Cl_2_, − 20 °C, 1 h.

At this point in the research, we wondered if the electrophilic reactivity of patulin could be exploited to achieve the pursued goal. Fliege and Metzler first suggested that antibody-based analytical methods for the detection of patulin might be feasible by analysing the adduct resulting from the reaction of the mycotoxin with a thiol-containing compound^23^. The determination of a derivative of the analyte instead of the analyte itself has already been used successfully for other very small chemicals such as glyphosate and acrylamide^32, 33^. For such an approach to be of practical interest, certain requirements must be met. Thus, (i) the reaction of patulin and the thiol should take place in an aqueous medium; (ii) the reaction should proceed through fast reaction kinetics so the product is formed within minutes rather than hours or days; (iii) the reaction should proceed reproducibly and in high yields, preferably quantitatively; and (iv) the reaction should result in a major compound, ideally a single one. While all of these requirements are important, the last one is of paramount relevance. Previous studies clearly showed that the reaction of patulin with thiols yields a large number of products through complex reaction pathways starting with a Michael-type addition to the electrophilic C-7 position of the α,β,γ,δ-unsaturated lactone. In particular, the reaction of patulin with 4-bromothiophenol allowed to identify up to sixteen different adducts^23^, far from the desirable goal of obtaining a single compound. However, careful examination of these results showed that five of the sixteen resulting adducts were formed because methanol was used as a co-solvent, while other products were formed under conditions where the thiol was present in high excess and/or after long reaction times. More importantly, these experiments also showed that one predominant compound was formed, namely the adduct formed by the incorporation of two thiol molecules into the mycotoxin scaffold at positions C-7 and C-4. Another key condition for the success of the derivatization strategy is that the final structure should be complex enough to elicit an appropriate immune response. Therefore, aromatic thiol-containing compounds seem to be more suitable as derivatization reagents than aliphatic thiols. Taking all these considerations into account, different reaction conditions were investigated to improve the formation of a di-thiol adduct in the absence of methanol and using six simple and commercially available aromatic thiols (see Fig. 5A for the structure of the chemicals used).

It was found that the di-thiol adduct was essentially the only product (as shown by thin layer chromatography and ^1^H NMR of the crude reaction mixture) when the reaction was carried out in 100 mM sodium phosphate buffer, pH 7.4, at a temperature between 0 °C and 25 °C, and using 2–3 equivalents of the sodium salt of the thiol, which was independently prepared by reaction of the thiol with one equivalent of sodium tert-butoxide. Therefore, under these reaction conditions, most of the assayed thiols led to the rapid and high-yield formation of the corresponding adduct, visible by the development of an intense yellow colour. This was particularly true for adducts **I** and **V**, whereas the formation of adduct **VI** was significantly less clean and lower yielding, most likely due to the high steric hindrance caused by the substitution of the two positions *ortho* to the thiolate group.

It was also observed that the di-thiol adduct was predominant even when one equivalent of the thiolate was used, which indicates that, once a thiolate group reacted with patulin, the incorporation of the second thiolate group to the initially formed mono-thiol adduct is favoured over the addition of the first one to an intact patulin molecule. Moreover, the di-thiol adduct was the major product even when excess thiolate was employed if relatively short incubation times were used (for example, 40 equivalents of thiol salt and 1 h of reaction time). These results were encouraging, opening up the possibility of attaining the goal of developing rapid point-of-need tests based on the derivatization of patulin with a thiolated compound. The adduct resulting from the reaction of patulin with 4-bromothiophenol (adduct **V**) was selected for the generation of antibodies because this compound is expected to possess good immunogenic properties due to the presence of two bromine atoms.

Because the patulin structure is opened upon reaction with two thiol molecules, the resulting adduct has a free carboxylic acid (Fig. 5), a beneficial result because it can be readily used for coupling the adduct to carrier proteins by carbodiimide-mediated chemistry. Nevertheless, direct conjugation of adduct **V** may mask the main antigenic determinants due to the close proximity of its molecular skeleton to the protein core, so an analogue of adduct **V** with a spacer arm was also synthesized (hapten **V4**; Fig. 5B). The carboxylic group in both derivatives, adduct **V** and hapten **V4**, was activated by forming the corresponding NHS esters. Thereafter, the compounds were conjugated to BSA and OVA and the corresponding bioconjugates were characterized by MALDI-TOF analysis. Adequate hapten densities of 10.7 and 16.0 were determined for conjugates BSA–**V** and BSA–**V4**, respectively (Fig. 4B), whereas values around 3 were estimated for OVA conjugates.

**Figure 6:**
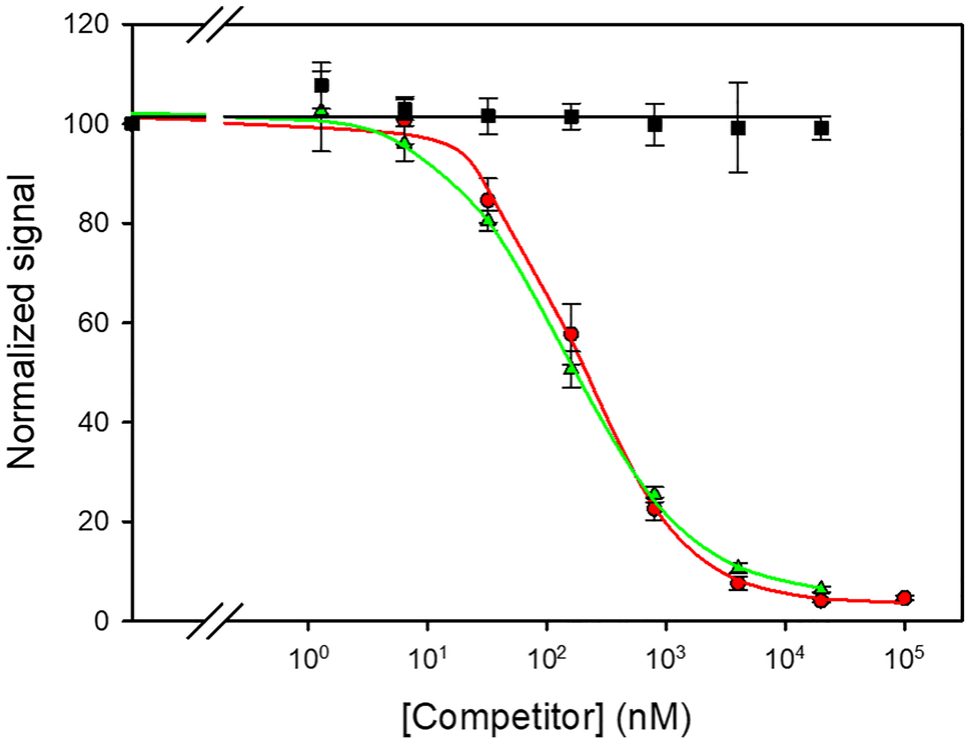
Inhibition curves with the antiserum that was derived from conjugate BSA-**V4**. *Assay conditions:* ELISA plates were coated with conjugate OVA‒**V4** (1 µg/mL), and the antibody was assayed at a 1/3,000 dilution. Competitors were patulin (squares), purified adduct **V** (circles), or a mixture between patulin and the sodium salt of 4-bromothiophenol (triangles).

Both BSA–**V** and BSA–**V4** conjugates were used to immunize rabbits, and the resulting antisera were assayed under standard conditions for competitive immunoassays. Each polyclonal antibody was able to recognize its corresponding homologous antigen, i.e., the antiserum from the rabbit immunized with conjugate BSA–**V** was able to bind to the immobilized conjugate OVA–**V**, and the antiserum from the rabbit immunized with conjugate BSA–**V4** recognized the coating antigen OVA–**V4**. The ability of these antisera to recognize the adduct **V** in solution was then examined. Standard curves of adduct **V** from 100 µM were prepared in PBS by five-fold serial dilution and added to coated plates, followed by adding the polyclonal antibody in PBST. Remarkably, both antisera were able to bind adduct **V**, as evidenced by the inhibition of the assay signal as the concentration of the analyte increased, while they were unable to recognize patulin or the derivatizing thiol. The polyclonal antibody from hapten **V4** exhibited a slightly higher affinity for the analyte than the antibody derived from immunizing with the adduct **V** (IC_50_ = 180 nM *vs* IC_50_ = 510 nM). This is most likely due to the presence of the spacer arm in hapten **V4,** which probably favours a better display of the molecule to the surrounding medium, and thus a better interaction with the antibody may occur. More importantly, when the thiolate at 2 mg/mL in water was added (2% (v/v)) to patulin solutions in buffer, and the mixtures were assayed by competitive ELISA after incubation for 1 h at room temperature, the resulting inhibition curve overlapped with the curve that was obtained with the purified adduct **V** (Fig. 6).

This result paves the way to develop user-friendly immunoanalytical procedures for the detection of patulin after a straightforward and rapid derivatization reaction. The detectability of the assay (35 nM), although certainly outstanding for such a small compound, is still lower than desirable for the development of an immunoassay enabling the reliable determination of patulin at the more challenging regulated level (10 µg/kg). However, it does mean an encouraging proof-of-concept that the electrophilic reactivity of patulin can be harnessed to generate suitable antibodies for food immunodiagnostics.

## Conclusions

Three functionalized derivatives of patulin that conformed to commonly accepted rules in hapten design for antibody production were synthesized in the present study. Unfortunately, these conventional approaches did not lead to the production of antibodies that could recognize free patulin in solution. In addition to the small size of the molecule, the high electrophilic reactivity of patulin is most likely responsible for these negative results. This disappointing outcome prompted us to explore a disruptive approach consisting of exploiting the high reactivity of patulin towards nucleophiles. Hence, antibodies against the adduct resulting from the reaction between patulin and a thiol-containing compound, 4-bromothiophenol, were obtained. Remarkably, the affinities of the antibodies thus generated for the patulin adduct were in the nanomolar range (K_D,app_ = 35 nM). Although there is still room for improvement, this strategy opens new avenues for future developments on antibody-based methods for sensitive immunodetection of this challenging mycotoxin. Further work should address innovative ways of preparing more rigid and antigenic adducts of patulin that might allow the generation of antibodies with even superior binding properties, for example using aromatic 1,2-dithiols instead of monothiols. If this can be achieved, the long-standing goal of monitoring patulin contamination by widely established immunochemical methods in food immunodiagnostics, such as ELISA, immunostrips, and immunoaffinity columns, will become a reality.

## Materials and methods

### Reagents and instruments

Chemicals and apparatus, general experimental techniques for hapten synthesis, details on the preparation of the conventional haptens **C4**, **C6**, and **C7**, and ^1^H NMR spectra of haptens, adducts, and NHS esters are provided as Supplementary Information.

### Preparation of patulin-dithiol adducts

*Preparation of (Z)-5-((4-bromophenyl)thio)-3-(((4-bromophenyl)thio)methylene)-6-hydroxy-4-oxohexanoic acid (adduct V).* A solution of 4-bromobenzenethiol (150 mg, 0.79 mmol) and sodium *tert*-butoxide (83.5 mg, 0.87 mmol, 1.1 equiv) in anhydrous MeOH was stirred at 50 °C for 1 h under nitrogen atmosphere. The solvent was eliminated under reduced pressure to afford sodium 4-bromobenzenethiolate as a white powder. A solution of patulin (10.3 mg, 0.067 mmol) in 8 mL of 100 mM sodium phosphate buffer (PB), pH 7.4, was cooled in an ice-water bath and a solution of the above sodium thiolate (44.1 mg, 0.194 mmol, 2.9 equiv) in Mili-Q H_2_O (5 mL) was added dropwise. The reaction mixture turned an intense yellow color almost immediately, which indicates how quickly the reaction is taking place. After 10–20 min stirring at the same temperature, the reaction mixture was acidified with formic acid to pH 3–4 and extracted with EtOAc (3 × 20 mL). The combined organic layers were washed with brine (15 mL), dried over anhydrous MgSO_4_, concentrated in vacuo, and finally purified by silica gel flash chromatography, using CHCl_3_–MeOH (95:5) with 0.1% of HCO_2_H as eluent, to afford adduct **V** as a yellow oil (19.2 mg, 54%). The spectroscopic data of adduct **V** were in complete agreement with those described previously by Metzler. IR *v*_max_ (cm^−1^) 3339 m, 1705 m, 1472 m, 1230w, 1112w, 1019 s, 814 m; ^1^H NMR (500 MHz, acetone-d_6_) δ 7.62 (d, *J* = 8.5 Hz, 2H, H-3 Ph and H-5 Ph), 7.50 (m, 4H, H-2′ Ph, H-6′ Ph, H-2 Ph and H-6 Ph), 7.49 (s, 1H, *H–C*=C3), 7.43 (d, *J* = 8.5 Hz, 2H, H-3′ Ph and H-5′ Ph), 4.30 (br t, *J* = 7.0 Hz, 1H, H-5), 4.00 (dd, *J* = 11.3, 7.5 Hz, 1H, H-6), 3.78 (dd, *J* = 11.3, 6.4 Hz, 1H, H′-6), 3.77 (d, *J* = 17.0 Hz, 1H, H-2), 3.71 (d, *J* = 17.4 Hz, 1H, H′-2); ^13^C NMR (125 MHz, acetone-d_6_) δ 194.4 (C-4), 172.7 (C-1), 148.8 (H*C* = C3), 138.0 (C-3), 136.9 (2C, C-3′ Ph and C-5′ Ph), 133.3 (2C, C-3 Ph and C-5 Ph), 133.0 (2C, C-2′ Ph and C-6’ Ph), 132.8 (2C, C-2 Ph and C-6 Ph), 132.0 (C-1′ Ph), 127.0 (C-1 Ph), 123.3 (C-4′ Ph), 122.5 (C-4 Ph), 62.3 (C-6), 54.2 (C-5), 38.2 (C-2); HRMS (ESI) *m/z* calcd for C_19_H_17_Br_2_O_4_S_2_ [M+H]^+^ 530.8930 (51.4%), 532.8909 (100%) and 534.8889 (48.6%), found 530.8910, 532.8889 and 534.8867.

### Preparation and spectroscopic data of other di-thiol adducts

The procedure followed for the reaction of patulin with the other aromatic thiols was similar to that described above for the preparation of adduct **V**. The most relevant data for each case are given below.

*(Z)-6-Hydroxy-4-oxo-5-(p-tolylthio)-3-((p-tolylthio)methylene)hexanoic acid (Adduct I).* This adduct (13 mg, 50%), a yellow oil, was obtained from a solution of patulin (10 mg, 0.065 mmol) in 100 mM PB (12.8 mL) and a solution of sodium 4-methylbenzenethiolate (20.1 mg, 0.138 mmol, 2.1 equiv) in Mili-Q H_2_O (6.4 mL) at rt. The EtOAc extract of the reaction mixture was purified by silica gel flash chromatography using CHCl_3_–MeOH (from 98:2 to 95:5) as eluent. IR *v*_max_ (cm^−1^) 3021 m, 1713 m, 1493 m, 1216 s; ^1^H NMR (300 MHz, CDCl_3_) δ 7.38 (d, *J* = 8.1 Hz, 2H, H-2′ Ph and H-6′ Ph), 7.27 (s, 1H, =C–H), 7.25 (d, *J* = 8.0 Hz, 2H, H-2 Ph and H-6 Ph), 7.19 (d, *J* = 7.9 Hz, 2H, H-3′ Ph and H-5′ Ph), 7.09 (d, *J* = 7.9 Hz, 2H, H-3 Ph and H-5 Ph), 4.10 (dd, *J* = 8.2, 4.7 Hz, 1H, H-5), 4.04 (dd, *J* = 10.9, 8.2 Hz, 1H, H-6), 3.88 (d, *J* = 17.6 Hz, 1H, H-2), 3.84 (dd, *J* = 10.9, 4.6 Hz, 1H, H′-6), 3.40 (d, *J* = 17.3 Hz, 1H, H′-2), 2.37 and 2.31 (each s, 3H each, 2xMe Ph); ^13^C NMR (125 MHz, CDCl_3_) δ 194.0 (C-4), 175.1 (C-1), 152.9 (H*C* = C3), 139.7 (C-4′ Ph), 138.8 (C-4 Ph), 135.5 (2C, C-3 Ph and C-5 Ph), 133.8 (C-1′ Ph), 131.2 (2C, C-3′ Ph and C-5′ Ph), 130.3 (2C, C-2′ Ph and C-6′ Ph), 130.1 (2C, C-2 Ph and C-6 Ph), 125.7 (C-1 Ph), 123.9 (C-3), 61.5 (C-6), 52.4 (C-5), 38.1 (C-2), 21.4 and 21.3 (*2xMe*Ph); HRMS calcd for C_21_H_23_O_4_S_2_ [M+H]^+^ 403.1032, found 403.1018.

*(Z)-5-((2,4-Dimethylphenyl)thio)-3-(((2,4-dimethylphenyl)thio)methylene)-6-hydroxy-4-oxohexanoic acid (Adduct II).* This adduct (5.8 mg, 46%), a yellow oil, was obtained from a solution of patulin (4.5 mg, 0.029 mmol)) in 100 mM PB (5.4 mL) and a solution of sodium 2,4-dimethylbenzenethiolate (10.3 mg, 0.064 mmol, 2.2 equiv) in Mili-Q H_2_O (5.4 mL) at rt. The EtOAc extract of the reaction mixture was purified by silica gel flash chromatography using CHCl_3_–MeOH 95:5 as eluent. IR *v*_max_ (cm^−1^) 3354 m, 3009 m, 2920 m, 2855 m, 1701 m, 1647 m, 1559 m, 1217 m, 1051 m, 954 m, 812 m, 751 s; ^1^H NMR (300 MHz, CDCl_3_) δ 7.34 (d, *J* = 7.8 Hz, 1H, H-6′ Ph), 7.24 (d, *J* = 8.1 Hz, 1H, H-6 Ph), 7.08–6.95 (m, 3H, H-3 Ph, H-3′ Ph and H–C=C3), 7.01 (d, *J* = 8.1 Hz, 1H, H-5 Ph), 6.91 (d, *J* = 7.8 Hz, 1H, H-5′ Ph), 4.16 (dd, *J* = 8.4, 4.8 Hz, 1H, H-5), 4.03 (dd, *J* = 11.3, 8.4 Hz, 1H, H-6), 3.84 (dd, *J* = 11.3, 4.8 Hz, 1H, H′-6), 3.69 (d, *J* = 17.3 Hz, 1H, H-2), 3.29 (d, *J* = 17.3 Hz, 1H, H′-2), 2.39, 2.35, 2.32 and 2.26 (each s, 12H, 4xMePh); ^13^C NMR (125 MHz, CDCl_3_) δ 194.4 (C-4), 174.3 (C-1), 152.6 (H*C*=C3), 142.2 (C-2′ Ph), 139.2 (C-2 Ph), 137.6 (C-4′ Ph), 136.2 (C-4 Ph), 131.4 (2C, C-3 Ph and C-3′ Ph), 131.2 (C-6′ Ph), 129.9 (C-1′ Ph), 127.7 (2C, C-5 Ph and C-6 Ph), 127.3 (C-5′ Ph), 125.8 (C-1 Ph), 123.3 (C-3), 61.7 (C-6), 52.2 (C-5), 21.1, 21.0, 20.9 and 20.8 (4x*Me*Ph); HRMS calcd for C_23_H_27_O_4_S_2_ [M+H]^+^ 431.1345, found 431.1346.

*(Z)-4,4'-((2-(Carboxymethyl)-5-hydroxy-3-oxopent-1-ene-1,4-diyl)bis(sulfanediyl)) dibenzoic acid (Adduct III).* This adduct (a *ca*. 4:1 mixture of *Z/E* isomers, 5.2 mg, 51%), a yellow oil, was obtained from a solution of patulin (3.4 mg, 0.022 mmol) in 100 mM PB (4.0 mL) and a solution of the 4-mercaptobenzoic acid (7.5 mg, 0,49 mmol, 2.2 equiv) in Mili-Q H_2_O (4 mL) at rt. The EtOAc extract of the reaction mixture was purified by C_18_-reversed-phase flash chromatography using MeOH–H_2_O 7:3 as eluent. IR *v*_max_ (cm^−1^) 3060 m, 2361 m, 1698 s, 1596 s, 1421 m, 1281 m; ^1^H NMR (300 MHz, acetone-*d*_6_) [only signals of major *Z*-isomer are given] δ 8.06 (d, *J* = 8.5 Hz, 2H, H-2 Ph and H-6 Ph), 7.96 (d, *J* = 8.4 Hz, 2H, H-2′ Ph and H-6′ Ph), 7.68 (d, *J* = 8.5 Hz, 2H, H-3 Ph and H-5 Ph), 7.65 (s, 1H, H-1′′), 7.60 (d, *J* = 8.5 Hz, 2H, H-3′ Ph and H-5′ Ph), 4.54 (dd, *J* = 7.7, 6.0 Hz, 1H, H-4′′), 4.09 (dd, *J* = 11.2, 7.7 Hz, 1H, H-5′′), 3.87 (dd, *J* = 11.2, 5.9 Hz, 1H, H′-5′′), 3.78 (s, 2H, H-1′′′); ^13^C NMR (75 MHz, *acetone*-*d*_6_) [only signals of major Z-isomer are given] δ 194.8 (C-3′′), 172.6 (C-2′′′), 167.0 and 166.9 (C-1 and C-1′), 147.4 (C-1′′), 144.1 (C-4 Ph), 140.2 (C-4′ Ph), 133.1 (C-1 Ph), 132.3–130.2 (8C, C-6 Ph, C-2 Ph, C-6′ Ph, C-2′ Ph, C-3 Ph, C-5 Ph, C-3′ Ph and C-5′ Ph), 130.6 (C-1′ Ph), 127.6 (C-2′′), 62.8 (C-5′′), 53.9 (C-4′′), 38.2 (C-1′′′); HRMS calcd for C_21_H_19_O_8_S_2_ [M+H]^+^ 463.0516, found 463.0516.

*(Z)-2,2'-(((2-(Carboxymethyl)-5-hydroxy-3-oxopent-1-ene-1,4-diyl)bis(sulfanediyl)) bis(4,1-phenylene))diacetic acid (Adduct IV).* This adduct (7.2 mg, 49%), a yellow oil, was obtained from a solution of patulin (4.6 mg, 0.030 mmol) in 100 mM PB (5.8 mL) and a solution of 2-(4-mercaptophenyl)acetic acid (10.1 mg, 0.06 mmol, 2 equiv) in a 20 mM aqueous solution of NaOH (5.8 mL, 0.116 mmol, 3.9 equiv) at rt. The ethyl acetate extract of the reaction mixture was purified by silica gel flash chromatography using CHCl_3_-MeOH 95:5, containing a 0.1% of HCO_2_H, as eluent. IR *v*_max_ (cm^−1^) 3021w, 1711s, 1363 m, 1221 s, 1093w; ^1^H NMR (500 MHz, acetone-d_6_) δ 7.51 (d, *J* = 8.2 Hz, 2H, H-2 Ph and H-6 Ph), 7.49 (s, 1H, H-1′′), 7.45 (d, *J* = 8.2 Hz, 2H, H-2′ Ph and H-6′ Ph), 7.39 (d, *J* = 8.4 Hz, 2H, H-3′ Ph and H-5′ Ph), 7.30 (d, *J* = 8.2 Hz, 2H, H-3 Ph and H-5 Ph), 4.26 (dd, *J* = 7.8, 6.1 Hz, 1H, H-4′′), 4.00 (dd, *J* = 11.1, 7.8 Hz, 1H, H-5′′), 3.79 and 3.71 (each d, *J* = 17.3 Hz, 2H, H-1′′′), 3.77 (dd, *J* = 11.1, 6.1 Hz, 1H, H-5′′), 3.68 and 3.64 (each s, 2H each, H-2′′ and H′-2′′); ^13^C NMR (125 MHz, acetone-d_6_) δ 194.6 (C-3′′), 172.8 (C-2′′′), 172.5 and 172.4 (C-1 and C-1′), 149.9 (C-1′′), 147.7 (C-4 Ph), 136.9, 136.5 and 136.1(3C, C-1 Ph, C-1′ Ph and C-4′ Ph), 135.0 (2C, C-6′ Ph and C-2′ Ph), 131.4 (2C, C-3′ Ph and C-5′ Ph), 131.3 (2C, C-6 Ph and C-2 Ph), 131.0 (2C, C-3 Ph and C-5 Ph), 126.5 (C-2′′), 62.4 (C-5′′), 54.3 (C-4′′), 40.8 and 40.7 (C-2 and C-2′), 38.3 (C-1′′′); HRMS calcd for C_23_H_23_O_8_S_2_ [M+H]^+^ 491.0829, found 491.0810.

*(Z)-6-Hydroxy-5-(mesitylthio)-3-((mesitylthio)methylene)-4-oxohexanoic acid (Adduct VI).* This adduct (3.0 mg, 18%), a yellow oil, was obtained from a solution of patulin (5.6 mg, 0.036 mmol) in 100 mM PB (6.7 mL) and a solution of the sodium salt of 2,4,6-trimethylbenzenethiol (13.8 mg, 0.079 mmol, 2.2 equiv) in 6.7 mL of Mili-Q H_2_O at rt. The EtOAc extract of the reaction mixture was purified by silica gel flash chromatography using CHCl_3_–MeOH 95:5 as eluent. As deduced from the analysis of the ^1^H NMR spectrum, this compound exists in solution as a mixture of two isomers, most probably rotamers around the C–S bonds. IR *v*_max_ (cm^−1^) 3330 m, 2922 m, 2855 m, 2100w, 1714s, 1671 m, 1619 m, 1602 m, 1457 m, 1399 m, 1375 m, 1164 s, 1034 m, 1012 m, 851 m, 754 s; ^1^H NMR (300 MHz, acetone-*d*_6_) [only signal of the major rotamer are given] δ 7.02 (s, 2H, H-3′ Ph and H-5′ Ph), 6.97 (s, 2H, H-3 Ph and H-5 Ph), 6.83 (br s, 1H, *H*–C=C3), 4.17 (m, 1H, H-5), 3.97 (dd, *J* = 10.7, 8.0 Hz, 1H, H-6), 3.75 (dd, *J* = 10.7, 5.3 Hz, 1H, H′-6), 3.27 and 3.05 (each d, *J* = 17.2 Hz, 1H each, H-2), 2.50 (s, 6H, Me*-*C2′ and Me-C6′), 2.38 (s, 6H, Me*-*C2 and Me*-*C6), 2.26 and 2.24 (each s, 3H each, Me*-*C4 and Me*-*C4′); HRMS calcd for C_16_H_19_O_4_S [M–Me_3_PhS]^+^ 307.0999, found 307.0998.

### Synthesis of hapten V4 [(Z)-3-(5-((4-bromophenyl)thio)-3-(((4-bromophenyl)thio)methylene)-6-hydroxy-4-oxohexanamido)propanoic acid]

A mixture of adduct **V** (19.2 mg, 0.036 mmol), EDC·HCl (9.7 mg, 0.051 mmol, 1.4 equiv) and NHS (5.8 mg, 0.051 mmol, 1.4 equiv) in CH_2_Cl_2_ (0.8 mL) was stirred at − 20 °C for 1 h. The solvent was eliminated under vacuum and the residue was dissolved in dry MeCN (0.4 mL). In a separate vial, β-alanine (19.2 mg, 0.216 mmol, 6.0 equiv) and NaHCO_3_ (18.1 mg, 0.216 mmol, 6.0 equiv) were dissolved in Mili-Q H_2_O (0.4 mL), this solution was added to the former, and the mixture was stirred for 1 h at 0 °C and 1 h at rt. The reaction mixture was cooled in an ice bath, acidified with citric acid, and extracted with EtOAc (3 × 20 mL). The combined organic layers were washed with brine (15 mL), dried over anhydrous MgSO_4_, concentrated in vacuo, and purified by silica gel flash chromatography, using CHCl_3_-MeOH (95:5) as eluent, to afford hapten **V4** as a light yellow oil (3.9 mg, 18%). IR *v*_max_ (cm^−1^) 3298 m, 1653w, 1448 m, 1230w, 1112w, 1019 s, 611 m; ^1^H NMR (300 MHz, acetone-d_6_) δ 7.61 (d, *J* = 8.4 Hz, 2H, H-3 Ph and H-5 Ph), 7.49 (m, 4H, H-2′′ Ph, H-6′′ Ph, H-2 Ph and H-6 Ph), 7.42 (d, *J* = 8.1 Hz, 2H, H-3′′ Ph and H-5′′ Ph), 7.42 (s, 1H, H–C=C3), 4.52 (br t, *J* = 7.0 Hz, 1H, H-5′), 4.00 (dd, *J* = 11.0, 7.8 Hz, 1H, H-6′), 3.76 (dd, *J* = 11.1, 6.2 Hz, 1H, H′-6′), 3.64 (d, *J* = 17.1 Hz, 1H, H-2′), 3.55 (d, *J* = 17.1 Hz, 1H, H′-2′) 3.43 (m, 2H, H-3), 2.50 (t, *J* = 6.3 Hz, 2H, H-2); ^13^C NMR (125 MHz, acetone-d_6_) δ 194.8 (C-4′), 172.2 (C-1), 169.3 (C-1′), 147.9 (H*C*=C3), 138.2 (C-3), 136.6 (2C, C-3′′ Ph and C-5’’ Ph), 133.2 (2C, C-3 Ph and C-5 Ph), 133.0 (2C, C-2’’ Ph and C-6’’ Ph), 132.8 (2C, C-2 Ph and C-6 Ph), 128.1 (C-1′′ Ph), 125.8 (C-1 Ph), 123.0 (C-4′′ Ph), 122.4 (C-4 Ph), 62.4 (C-6′), 53.9 (C-5′), 40.3 (C-3), 36.2 (C-2′), 34.4 (C-2); HRMS (ESI) *m/z* calcd for C_22_H_22_Br_2_NO_5_S_2_ [M+H]^+^ 601.9301 (51.4%), 603.9280 (100%) and 605.9260 (48.6%), found 601.9298, 603.9274 and 605.9270.

### Activation of adduct V and hapten V4 with NHS by carbodiimide-mediated chemistry

*Preparation of 2,5-dioxopyrrolidin-1-yl (Z)-5-((4-bromophenyl)thio)-3-(((4-bromophenyl) thio)methylene)-6-hydroxy-4-oxohexanoate (V–NHS).* Adduct **V** (10.5 mg, 0.020 mmol), NHS (2.8 mg, 0.024 mmol, 1.2 equiv) and EDC∙HCl (5.0 mg, 0.026 mmol, 1.3 equiv) were dissolved in of anhydrous CH_2_Cl_2_ (0.5 mL) and stirred for 1 h at − 20 °C under nitrogen. It was observed that if the formation of the active ester is carried out at higher temperatures, the OH at C-6 is eliminated, resulting in the formation of a terminal double bond between C-5 and C-6. For example, the active ester of the mentioned elimination product is formed mostly at room temperature. Upon completion, the reaction mixture was diluted with CH_2_Cl_2_ and washed successively with water and brine, and then dried over anhydrous MgSO_4_. Evaporation of the solvent under reduced pressure afforded compound **V–NHS** (11.5 mg, 79%) as a brownish oil. ^1^H NMR (300 MHz, CDCl_3_) δ 7.54 (d, *J* = 8.6 Hz, 2H, H-3 Ph and H-5 Ph), 7.43 (d, *J* = 8.7 Hz, 2H, H-3′ Ph and H-5′ Ph), 7.41 (s, 1H, H–C=C3), 7.37 (d, *J* = 8.6 Hz, 2H, H-2 Ph and H-6 Ph), 7.26 (d, *J* = 8.7 Hz, 2H, H-2′ Ph and H-6′ Ph), 4.13 (dd, *J* = 7.8, 5.1 Hz, 1H, H-5), 4.06 (dd, *J* = 17.7, 0.9 Hz, 1H, H-2), 4.05 (dd, *J* = 11.2, 7.8 Hz, 1H, H-6), 3.87 (dd, *J* = 11.2, 5.1 Hz, 1H, H′-6), 3.76 (d, *J* = 17.7 Hz, 1H, H′-2), 2.85 (s, 4H, CO(CH_2_)_2_CO).

*Preparation of 2,5-dioxopyrrolidin-1-yl (Z)-3-(5-((4-bromophenyl)thio)-3-(((4-bromophenyl)thio)methylene)-6-hydroxy-4-oxohexanamido)propanoate (V4–NHS).* Hapten **V4** (8.6 mg, 0.014 mmol), NHS (2.3 mg, 0.020 mmol, 1.4 equiv) and EDC∙HCl (3.8 mg, 0.020 mmol, 1.4 equiv) were dissolved in anhydrous CH_2_Cl_2_ (0.5 mL) and stirred for 1 h at − 20 °C under nitrogen atmosphere. Upon completion, the reaction mixture was diluted with CH_2_Cl_2_ and washed successively with water and brine, and then dried over anhydrous MgSO_4_. Evaporation of the solvent under reduced pressure afforded compound **V4–NHS** (5.5 mg, 55%) as a brownish oil. ^1^H NMR (300 MHz, acetone-d_6_) δ 7.62 (d, *J* = 8.7 Hz, 2H, H-3 Ph and H-5 Ph), 7.51 (d, *J* = 8.7 Hz, 2H, H-3′′ Ph and H-5′′ Ph), 7.50 (d, *J* = 8.7 Hz, 2H, H-2 Ph and H-6 Ph), 7.49 (s, 1H, H–C=C3), 7.42 (d, *J* = 8.7 Hz, 2H, H-2′′ Ph and H-6′′ Ph), 4.48 (dd, *J* = 7.8, 6.2 Hz, 1H, H-5′), 4.00 (dd, *J* = 11.1, 7.8 Hz, 1H, H-6′), 3.76 (dd, *J* = 11.1, 6.2 Hz, 1H, H′-6′), 3.66 (d, *J* = 17.1 Hz, 1H, H-2′), 3.57 (d, *J* = 17.1 Hz, 1H, H′-2′), 3.56 (m, 2H, H-3), 2.88 (t, *J* = 6.0 Hz, 2H, H-2), 2.87 (s, 4H, CO(CH_2_)_2_CO).

### Preparation of bioconjugates

Following activation, hapten coupling was carried out by dropwise adding a 50 mM solution of the active ester in *N,N*-dimethylformamide (or MeCN) to a 15 mg/mL BSA or OVA solution in 100 mM phosphate buffer, pH 7.4, under gentle stirring overnight at room temperature. BSA–hapten immunizing conjugates were prepared by employing 30 (haptens **C4**, **C6** and **C7**), 40 (hapten **V4**) or 60 (adduct **V**) mol of activated hapten per mol of protein, whereas for OVA–hapten assay conjugates 10 (haptens **C4**, **C6** and **C7**) or 15 (adduct **V** and hapten **V4**) equivalents were used. Conjugates were purified by size exclusion chromatography using three connected 5 mL Sephadex G-25 HiTrap desalting columns and using 100 mM phosphate buffer, pH 7.4, as eluent. BSA conjugates were filter-sterilized through 0.45 μm pore filters and stored at − 20 °C. Final hapten-to-protein molar ratios were determined by Matrix-Assisted Laser Desorption Ionization Time-of-Flight Mass Spectrometry (MALDI-TOF/MS).

### MALDI mass spectrometry analysis of bioconjugates

Analysis of hapten bioconjugates was performed by mass spectrometry as follows:

*Sample preparation.* 100 μL of protein conjugate (0.5‒1 mg/mL) were dialyzed against milliQ water and lyophilized. The sample was dissolved in MilliQ water to a theoretical final concentration of 1 mg/mL, and 1 μL was spotted onto the MALDI plate. After the droplet was air-dried at room temperature, 1 μL of matrix (10 mg/mL sinapinic acid (Bruker) in 70% MeCN, 0.1% Trifluoroacetic acid) was added and allowed to air-dry at room temperature.

*Mass spectrometry analysis.* The sample was analyzed in a 5800 MALDI TOF/TOF (ABSciex) instrument in positive linear mode (1500 shots every position) in a mass range of 15,000‒100,000 m/z. Previously, the plate was calibrated with 1 μL of the TOF/TOF calibration mixture (ABSciex), in 13 positions. Every sample was calibrated by ‘close external calibration’ method with a BSA or OVA spectrum acquired in a close position. The analysis of the results was performed using the mMass program (http://www.mmass.org/).

### Animal immunization

Experimental design was approved by the Bioethics Committee of the University of Valencia (procedure number 2019/VSC/PEA/0179). The study was carried out in compliance with the ARRIVE guidelines. Animal manipulation was performed in compliance with the European Directive 2010/63/EU and the Spanish laws and guidelines (RD1201/2005 and 32/2007) concerning the protection of animals used for scientific purposes. Female New Zealand white rabbits were immunized by subcutaneous injection with 300 μg of the corresponding BSA–hapten conjugate. The immunogen consisted of a 1:1 emulsion between the conjugate in PBS and Freund’s adjuvant (complete for the first inoculation and incomplete for subsequent boosts). Four injections were applied at three-week intervals, followed by animal exsanguination by intracardiac puncture ten days after the last injection. Blood samples were left to coagulate at 4 °C overnight and the serum was separated by centrifugation. Antibodies were precipitated twice with a saturated ammonium sulfate solution in water and stored at 4 °C.

### Evaluation of the immune response

Immunoassays were carried out in the indirect ELISA format. Microtiter plates were coated by adding 100 µL per well of the corresponding OVA–hapten conjugate in 50 mM carbonate-bicarbonate buffer, pH 9.6. After overnight incubation at room temperature, the plates were washed four times with a 150 mM NaCl solution. The competitive assay was carried out by adding 50 μL per well of the analyte in PBS plus 50 μL per well of antiserum solution in PBST (PBS containing 0.05% (v/v) Tween 20), and the plates were incubated one hour at room temperature. After plate washing, 100 μL per well of the enzyme-labeled secondary antibody (GAR–HRP (1/10000)) in PBST containing 10% (v/v) fetal bovine serum was added and incubated at room temperature for an additional hour. The plates were washed again and the signal was generated by adding 100 µL per well of TMB enzyme substrate solution. Following incubation during 10 min at room temperature, the reaction was stopped by adding 100 µL per well of 1 M H_2_SO_4_, and the absorbances at 450 nm were immediately read using a reference wavelength at 650 nm. The experimental values were fitted to a four-parameter logistic equation.

## Supplementary Information

Supplementary Information.

## Data Availability

Upon signing of a material transfer agreement, limited amounts of the materials herein described are available for evaluation upon request to the corresponding authors.
