# Supplementary material for: Chemical strategies for triggering the immune response to the mycotoxin patulin
Source: Sci Rep. 2021 Dec 6;11:23438. doi: 10.1038/s41598-021-02916-6 (PMC8648828; doi:10.1038/s41598-021-02916-6)

SUPPLEMENTARY INFORMATION

Chemical strategies for triggering the immune response to the mycotoxin patulin

Hadyn Duncan, Josep V. Mercader, Consuelo Agulló, Marcos Gil-Sepulcre,

Antonio Abad-Somovilla, Antonio Abad-Fuentes

Contents **Page**

1. General experimental procedures and techniques S-2

2. Synthesis of haptens **C4** and **C7** S-3

3. Synthesis of hapten **C6** S-9

4. Hapten Activation with NHS by carbodiimide-mediated chemistry S-18

5. References S-19

6. Copies of ^{1}H NMR spectra of haptens, adducts, and NHS esters S-20

1. General experimental procedures and techniques

1.1. Reagents, equipment, and general techniques in synthesis procedures

Benzene and tetrahidrofurano (THF) were distilled over Na and benzophenone under nitrogen atmosphere just before use. CH_2Cl_2 and CH_3CN were distilled from CaH_2 in the same way.[1] Anhydrous *N,N*-dimethylformamide (DMF), MeOH, and acetone were purchased from Fisher Scientific (Madrid, Spain). Patulin was purchased from Fermentek (Jerusalem, Israel). The remaining solvents and commercial reagents were used without prior purification. The operations with air and/or moisture-sensitive reagents were carried out under an inert atmosphere of dry nitrogen, using syringes or cannulas, oven-dried (140 °C) glass material, and freshly distilled and dried solvents. Reactions were monitored by thin-layer chromatography on precoated silica plates (0.25 mm layer thickness, Silica Gel 60 F_{254}) using UV light as the visualizing agent and ethanolic phosphomolybdic acid or aqueous ceric ammonium molybdate solutions and heat as developing agents. The synthesized compounds were purified by flash column chromatography using silica gel 60 (particle size 0.043–0.063 mm). Melting points (Mp) were determined on a Büchi M-560 apparatus and are uncorrected. Optical rotations were recorded on a Perkin Elmer Mod. 343 polarimeter at a temperature of 20 °C, using a 1 dm cell and the solvent specified in each case; concentrations of the solutions are expressed in g/100 mL. IR spectra were recorded using a Nicolet Avatar 320 FT-IR spectrophotometer equipped with ATR (IR band intensities: w = weak, m = medium, s = strong). $^{1}H/^{13}C$ NMR spectra were recorded at 298 ^{o}K, in the solvent indicated, at 300/75 MHz (Bruker Avance DPX300 spectrometer) or 500/125 MHz (Bruker Avance DRX500). The chemical shifts are expressed in ppm (δ scale) relative to the residual solvent as the internal reference in all cases [7.27/77.00 ppm, 2.05/29.84 ppm and 3.31/49.00 ppm for the $^{1}H/^{13}C$ spectra in $CDCl_3$, acetone-d_6 and methanol-d_4, respectively. Carbon substitution degrees were established by DEPT pulse sequences. Complete assignment of ^{1}H and ^{13}C chemical shifts of selected compound was made based on a combination of COSY, HSQC and NOESY experiments. High-resolution mass spectra (HRMS) were obtained by electrospray ionization (ESI) mode in a premier Q-TOF mass spectrometer equipped with an electrospray source (Waters, Manchester, UK). The obtained data are expressed as mass/charge ratio (*m/z*).

1.2. Reagents and equipment used in antibody generation and immunoassays

BSA fraction V was from Roche Applied Science (Mannheim, Germany). OVA, adult bovine serum, Freund's adjuvants, and *o*-phenylenediamine were provided by Sigma/Aldrich (Madrid, Spain). Peroxidase-labeled goat anti-rabbit immunoglobulins antibody conjugate (#1706515)

was obtained from Bio-Rad Laboratories Inc. (Madrid, Spain). Costar flat-bottom high-binding 96-well polystyrene ELISA plates from Corning (Corning, NY, USA) were used. ELISA absorbances were read with a PowerWave HT from BioTek Instruments (Winooski, VT, USA). Microplate wells were washed with an ELx405 microplate washer also from BioTek Instrument.

2. Synthesis of haptens C4 and C7

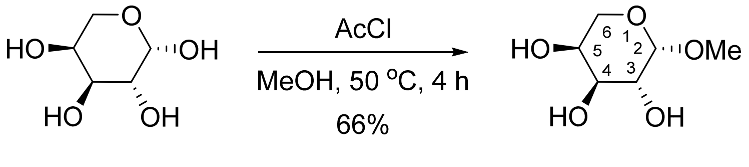

1 **2**

2.1. Preparation of (2*S*,3*R*,4*S*,5*S*)-2-methoxytetrahydro-2H-pyran-3,4,5-triol (2). Acetyl chloride (2 mL, 28 mmol, 2.1 equiv) was added dropwise to 70 mL of dry methanol in an ice bath and stirred for 30 minutes. Once rt was reached, L-(+)-arabinose (**1**) (9.0 g, 60 mmol) was added and the mixture was stirred at 50 °C for 4 hours. The mixture was then cooled to 0 °C for 1 hour allowing product **2** to crystalize. The obtained crystals were filtered and washed with cold methanol to afford **2** (6.49 g, 66%) as white crystals. Mp 165-168°C (MeOH) [Lit.[2] 166-168°C]; $[\alpha]_D$= +235° (c 1.1, MeOH); IR ν_{max} (cm^{-1}) 3355s, 2491m, 1652w, 1456w, 1198w, 1142w, 1064w, 998w; ^{1}H NMR (300 MHz, D_2O) δ 4.84 (d, *J* = 3.0 Hz, 1H, H-2), 4.00 (m, 1H, H-4), 3.88 (dd, *J* = 15.0, 3.0 Hz, 1H, H-6), 3.85 (m, 2H, H-3 and H-5), 3.66 (dd, *J* = 15.0, 3.0 Hz, 1H, H'-6), 3.42 (s, 3H, MeO); ^{13}C NMR (75 MHz, D_2O) δ 100.3 (C-2), 69.3 (C-4), 69.2 (C-3), 68.5 (C-5), 62.9 (C-6), 55.6 (MeO); HRMS (ESI) *m/z* calcd for $C_6H_{12}NaO_5$ $[M+Na]^{\cdot+}$ 187.0582, found 187.0582.

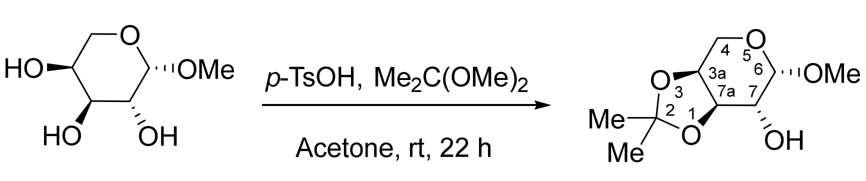

2 96% **3**

2.2. Preparation of (3a*S*,6*S*,7*R*,7a*R*)-6-methoxy-2,2-dimethyltetrahydro-3aH-[1,3]dioxolo[4,5-c]pyran-7-ol (3). Compound **2** (2.0 g, 12.12 mmol) and *p*-toluenesulfonic acid (5.2 mg, 0.028 mmol, 0.002 equiv) were dissolved in dry acetone (32 mL). 2,2-Dimethoxypropane (1.56 mL, 12.74 mmol, 1.05 equiv) was added and the mixture was stirred for 22 hours at rt. The mixture was then concentrated under reduced pressure, dissolved in EtOAc, washed with a 5% aqueous solution of $NaHCO_3$, brine, dried over anhydrous $MgSO_4$,

concentrated in vacuo, and finally purified by silica gel flash chromatography, using $CHCl_3$ as eluent, to afford **3** (2.39 g, 96%) as a clear oil. $[\alpha]_D$= +213° (c 1.1, $CHCl_3$) {Lit.[3] $[\alpha]_D$= +218.8°}; IR ν_{max} (cm^{-1}) 3383m, 2941m, 1732w, 1447w, 1359w, 1192w, 1137w, 1061m, 995w, 941w, 882w, 837w, 773w, 702w; ^1H NMR (300 MHz, $CDCl_3$) δ 4.71 (dd, *J* = 3.6, 0.6 Hz, 1H, H-6), 4.21 (dt, *J* = 5.8, 1.9 Hz, 1H, H-3a), 4.17 (dd, *J* = 6.8, 5.8 Hz, 1H, H-7a), 3.92 (m, 2H, H-4), 3.76 (ddd, *J* = 7.3, 6.8, 3.6 Hz, 1H, H-7), 3.43 (s, 3H, MeO), 2.35 (d, *J* = 7.3 Hz, 1H, OH), 1.54 and 1.37 (each s, each 3H, Me_2C); ^{13}C NMR (75 MHz, $CDCl_3$) δ 109.4 (C-2), 98.9 (C-6), 76.2 (C-3a), 76.1 (C-7a), 70.3 (C-7), 59.4 (C-4), 55.8 (MeO), 28.1 and 26.1 (Me_2C); HRMS (ESI) *m/z* calcd for $C_9H_{16}NaO_5$ $[M+Na]^{\cdot+}$ 277.0895, found 227.0890.

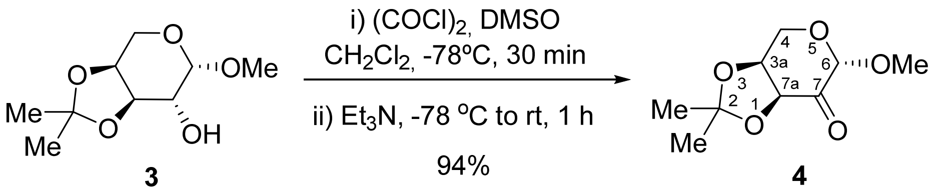

2.3. Preparation of (3a*S*,6*S*,7a*S*)-6-methoxy-2,2-dimethyldihydro-3aH-[1,3]dioxolo[4,5-c]pyran-7(4H)-one (4). DMSO (0.98 mL, 13.85 mmol, 2.8 equiv) in anhydrous CH_2Cl_2 (8.4 mL) was added dropwise over 5 min to a solution of $(COCl)_2$ (0.59 mL, 6.92 mmol, 1.4 equiv) in CH_2Cl_2 (11.5 mL) and the mixture was stirred for 15 minutes at −78 °C. Compound **3** (1.00 g, 4.95 mmol) in CH_2Cl_2 (11.5 mL) was then added dropwise and the reaction mixture was stirred for 30 min, followed by the addition of Et_3N (4.12 mL, 29.67 mmol, 6 equiv). The mixture was then stirred for a further 10 min at −78 °C, and then allowed to warm to rt and stirred for 1 hour. The mixture was diluted with H_2O and the aqueous layer was extracted with CH_2Cl_2. The combined organic layers were washed with brine, dried over anhydrous $MgSO_4$, concentrated in vacuo, and purified by silica gel flash chromatography, using $CHCl_3$-MeOH (95:5) as eluent, to afford ketone **4** (0.94 g, 94%) as a yellowish solid. Mp 93-94°C ($CHCl_3$) [Lit.[4] 88-91°C]; $[\alpha]_D$= +166° (c 0.4, $CHCl_3$); IR ν_{max} (cm^{-1}) 2986m, 2936m, 1751s, 1662w, 1455w, 1382w, 1224w, 1159w, 1119w, 1074s, 1029m, 969w, 891w, 868w, 847w, 784w, 766w; ^1H NMR (300 MHz, $CDCl_3$) δ 4.69 (s, 1H, H-6), 4.67 (d, *J* = 5.6 Hz, 1H, H-7a), 4.52 (ddd, *J* = 5.6, 2.1, 0.9 Hz, 1H, H-3a), 4.22 (dd, *J* = 13.4, 2.1 Hz, 1H, H-4), 4.06 (dd, *J* = 13.4, 0.9 Hz, 1H, H'-4), 3.48 (s, 3H, MeO), 1.45 and 1.38 (each s, each 3H, Me_2C); ^{13}C NMR (75 MHz, $CDCl_3$) δ 198.9 (C-7), 110.6 (C-2), 101.3 (C-6), 78.1 (C-7a), 75.8 (C-3a), 58.8 (C-4), 52.1 (MeO), 27.6 and 26.5 (Me_2C); HRMS (ESI) *m/z* calcd for $C_9H_{14}O_5$ $[M+H]^{\cdot+}$ 203.0875, found 203.0914.

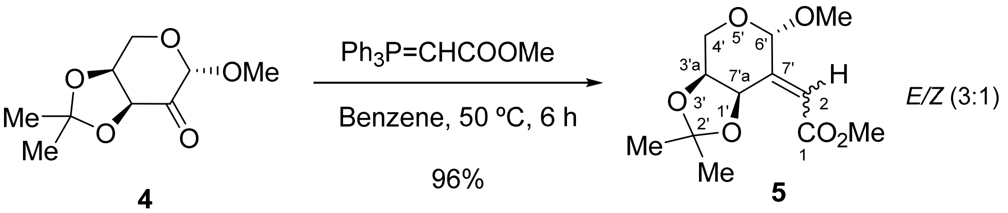

2.4. Preparation of methyl 2-((3aS,6S,7aR)-6-methoxy-2,2-dimethyl-3aH-[1,3]dioxolo[4,5-c]pyran-7(4H,6H,7aH)-ylidene)acetate (5). Ketone **4** (727.0 mg, 3.60 mmol) and methyl 2-(triphenylphosphoranylidene)acetate (1.36 g, 4.07 mmol, 1.1 equiv) were suspended in dry benzene (12 mL) under nitrogen. The mixture was heated at 50 °C and stirred for 6 hours. The resulting mixture was concentrated in vacuo and purified by silica gel flash chromatography, using hexane/EtOAc (8:2) as eluent, to afford unsaturated methyl ester **5** (880 mg, 96%) as a yellowish oil, a 3:1 mixture of *E/Z* stereoisomers. IR ν_{max} (cm^{-1}) 2985w, 2935w, 2828w, 1721s, 1669w, 1435w, 1380w, 1320w, 1244w, 1152w, 1128w, 1066w, 1038w, 1013w, 986w, 961w, 936w, 908w, 875w, 850w; ^{1}H NMR (300 MHz, $CDCl_3$) (only signals of the predominant *E*-isomer are given) δ 6.35 (dd, *J* = 1.8, 0.7 Hz, 1H, H-2), 6.02 (dt, *J* = 7.5, 0.5 Hz, 1H, H-7'a), 5.23 (dd, *J* = 1.9, 0.4 Hz, 1H, H-6'), 4.32 (dt, *J* = 7.5, 1.7 Hz, 1H, H-3'a), 3.75 (s, 3H, MeO_2C-1), 3.64 (d, *J* = 1.7 Hz, 2H, H-4'), 3.47 (s, 3H, MeO), 1.53 and 1.40 (each d, *J* = 0.7 Hz, 3H each, Me_2C); ^{13}C NMR (75 MHz, $CDCl_3$) (only signals of the predominant *E*-isomer are given) δ 166.0 (C-1), 148.2 (C-7'), 123.7 (C-2), 110.6 (C-2'), 97.8 (C-6'), 75.2 (C-3'a), 68.6 (C-7'a), 63.1 (C-4'), 55.6 (MeO), 51.9 ($\mathit{Me}O_2C$-1), 26.5 and 25.4 (Me_2C); HRMS (ESI) *m/z* calcd for $C_{12}H_{17}NaO_6$ $[M+Na]^{·+}$ 281.0996, found 281.1007.

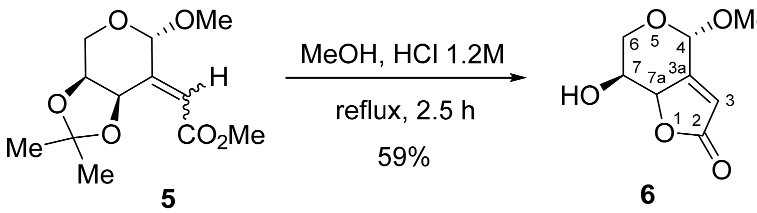

2.5. Preparation of (4*S*,7*S*,7a*R*)-7-hydroxy-4-methoxy-4,6,7,7a-tetrahydro-2H-furo[3,2-c]pyran-2-one (6). The mixture of α,β-unsaturated esters **5** (313 mg, 1.21 mmol) and 2,6-di-*tert*-butyl-4-methylphenol (25.1 mg, 0.11 mmol, 0.1 equiv) were dissolved in anhydrous methanol (13 mL) under nitrogen. Aqueous 1.2 M HCl solution (300 µL, 0.36 mmol, 0.3 equiv) was then added and the mixture was stirred at reflux for 2.5 hours. The reaction mixture was cooled to rt and then extracted with CH_2Cl_2, washed with brine, dried over anhydrous $MgSO_4$, concentrated in vacuo, and purified by silica gel flash chromatography, using hexane-EtOAc (1:1) as eluent, to afford hydroxy γ-lactone **6** (132.2 mg, 59%) as a white solid. Mp 126-127°C ($CHCl_3$) [Lit.[44] 134-

136^{oC}]; $[\alpha]_D$= +236° (c 0.9, $CHCl_3$); IR ν_{max} (cm^{-1}) 3446s, 3106m, 2936s, 1784m, 1747s, 1436w, 1346w, 1312w, 1230w, 1193m, 1142m, 1094m, 1051m, 1023m, 971w, 891m, 841w, 801w; ^{1}H NMR (300 MHz, $CDCl_3$) δ 5.99 (dd, *J* = 1.9, 0.8 Hz, 1H, H-3), 5.48 (d, *J* = 0.7 Hz, 1H, H-4), 5.13 (ddd, *J* = 4.1, 1.9, 0.6 Hz, 1H, H-7a), 4.36 (t, *J* = 4.2 Hz, 1H, H-7), 3.99 (d, *J* = 12.7 Hz, 1H, H-6), 3.84 (dd, *J* = 12.7, 2.3 Hz, 1H, H'-6), 3.48 (s, 3H, MeO); ^{13}C NMR (75 MHz, $CDCl_3$) δ 172.4 (C-2), 159.9 (C-3a), 115.5 (C-3), 95.6 (C-4), 78.3 (C-7a), 69.7 (C-7), 61.3 (C-6), 55.5 (MeO); HRMS (ESI) *m/z* calcd for $C_8H_{11}O_5$ $[M+H]^{\cdot+}$ 187.0601, found 187.0594.

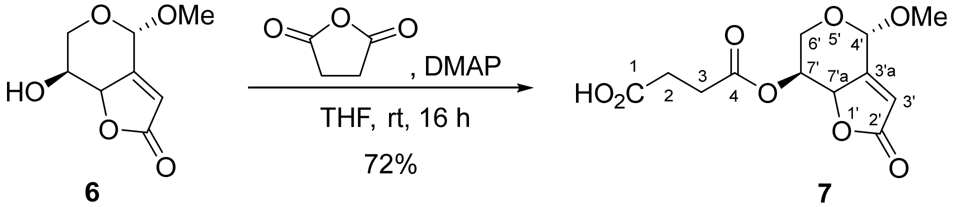

2.6. Preparation of 4-(((4*S*,7*S*,7a*R*)-4-methoxy-2-oxo-4,6,7,7a-tetrahydro-2H-furo[3,2-c]pyran-7-yl)oxy)-4-oxobutanoic acid (7). Alcohol **6** (10 mg, 0.054 mmol) was dissolved in dry THF (0.3 mL) under nitrogen. Succinic anhydride (53.8 mg, 0.537 mmol, 10 equiv) and 4-dimethylaminopyridine (0.7 mg, 6 µmol, 0.1 equiv) were dissolved in of dry THF (0.6 mL) and added dropwise. The mixture was stirred 20 hours at rt before being diluted with H_2O (2 mL) and extracted with $CHCl_3$. The combined organic layers were then washed with H_2O, brine, dried over $MgSO_4$, and concentrated in vacuo. The residue obtained was purified by chromatography, using $CHCl_3$-MeOH (95:5) as eluent, to obtain acid **7** (11 mg, 72%) as an amorphous white solid. IR ν_{max} (cm^{-1}) 2937w, 1788m, 1743s, 1413m, 1382w, 1348m, 1195m, 1164m, 1054m, 1100m, 975m, 862w, 800w; ^{1}H NMR (300 MHz, $CDCl_3$) δ 6.02 (dd, *J* = 2.0, 0.8 Hz, 1H, H-3'), 5.51 (s, 1H, H-4'), 5.49 (ddd, *J* = 4.0, 1.8, 1.0 Hz, 1H, H-7'), 5.20 (dd, *J* = 4.3, 1.9 Hz, 1H, H-7'a), 4.01 (dt, *J* = 13.1, 0.8 Hz, 1H, H-6'), 3.87 (dd, *J* = 13.3, 2.2 Hz, 1H, H'-6'), 3.49 (s, 3H, MeO), 2.74-2.53 (m, 4H, H-2 and H-3); ^{13}C NMR (75 MHz, $CDCl_3$) δ 176.8 (C-1), 171.8 (C-4), 171.1 (C-2'), 159.2 (C-3'a), 115.3 (C-3'), 95.5 (C-4'), 75.5 (C-7'a), 70.5 (C-7'), 59.3 (C-6'), 55.6 (MeO), 29.0 and 28.8 (H-2 and H-3); HRMS (ESI) *m/z* calcd for $C_{12}H_{18}NO_8$ $[M+NH_4]^{\cdot+}$ 287.0761, found 287.0766.

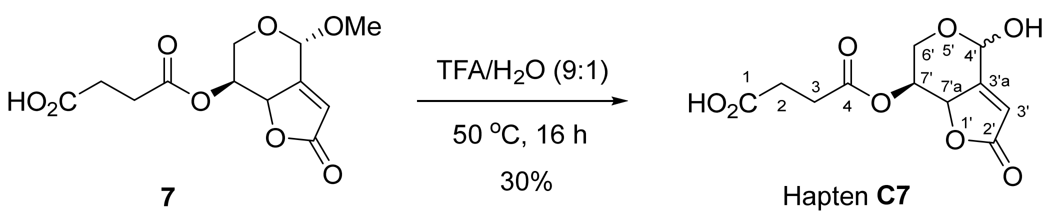

2.7. Preparation of 4-(((4*S*,7*S*,7a*R*)-4-hydroxy-2-oxo-2,6,7,7a-tetrahydro-4H-furo[3,2-c]pyran-7-yl)oxy)-4-oxobutanoic acid (Hapten C7). Compound **7** (8.4 mg, 0.029 mmol) was dissolved in a 9:1 mixture of trifluoroacetic acid-H_2O (0.6 mL) and stirred at 45-50 °C overnight. The mixture was then concentrated in vacuo and purified by silica gel flash chromatography, using $CHCl_3$-isopropyl alcohol (95:5) as eluent, to afford hapten **C7** (2.4 mg, 30%) as clear film. As observed by ^1H NMR, the product was an approximately 7:3 mixture of the two hemiacetal epimers at C-4′, in equilibrium with a small percentage of the open hydroxy-aldehyde form. IR ν_{max} (cm^{-1}) 3404w, 1735s, 1416w, 1161m, 1054m, 1010m; ^1H NMR (300 MHz, acetone d_6) [only signal of the major epimer are given] δ 6.07 (dd, *J* = 2.0, 0.8 Hz, 1H, H-3′), 6.06 (s, 1H, H-4′), 5.53 (ddd, *J* = 4.4, 2.2, 0.9 Hz, 1H, H-7′), 5.44 (ddd, *J* = 4.5, 1.9, 0.5 Hz, 1H, H-7′a), 4.33 (ddd, *J* = 13.3, 1.0, 0.6 Hz, 1H, H-6′), 3.78 (dd, *J* = 13.3, 2.3 Hz, 1H, H′-6′), 2.58-2.55 (m, 4H, H-2 and H-3); ^{13}C NMR (125 MHz*,* $CDCl_3$) [only signal of the major epimer are given] δ 173.7 (C-1), 173.2 (C-4), 171.9 (C-2′), 162.8 (C-3′a), 114.1 (C-3′), 89.6 (C-4′), 76.1 (C-7′), 71.7 (C-7′a), 59.6 (C-6′), 29.0 (2C, C-2 and C-3); HRMS (ESI) *m/z* calcd for $C_{11}H_{13}O_8$ $[M+H]^{\cdot+}$ 273.0605, found 273.0604.

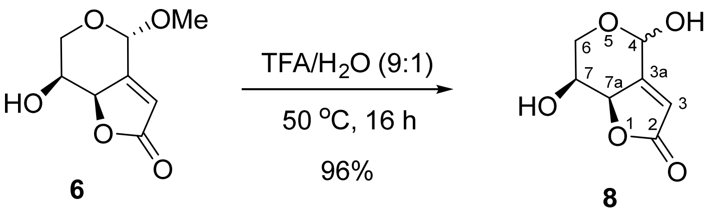

6 8

2.8. Preparation of (4*S*,7*S*,7a*R*)-7-hydroxy-4-methoxy-4,6,7,7a-tetrahydro-2H-furo[3,2-c]pyran-2-one (8). Compound **6** (9.4 mg, 0.051 mmol) was dissolved in a mixture 9:1 of trifluoroacetic acid-H_2O and stirred overnight at 50 °C. The mixture was then concentrated in vacuo and purified by silica gel flash chromatography, using $CHCl_3$-MeOH (95:5) as eluent, to afford **8** (8.6 mg, 96%) as a white solid.[5] IR ν_{max} (cm^{-1}) 3356s, 1737s, 1672s, 1452w, 1201s, 1141s, 1016m, 847w, 804m, 728 m; ^1H NMR (300 MHz, acetone-d_6) δ 5.95 (dd, *J* = 1.9, 0.8 Hz, 1H, H-3), 5.94 (m, 1H, H-4), 5.24 (ddd, *J* = 4.1, 1.9, 0.6 Hz, 1H, H-7a), 4.34 (ddt, *J* = 4.0, 2.4, 0.9 Hz, 1H, H-7), 4.23 (ddd, *J* = 12.6, 1.1, 0.6 Hz, 1H, H-6), 3.69 (dd, *J* = 12.5, 2.4 Hz, 1H, H′-6); ^{13}C NMR (75 MHz*,* $CDCl_3$) δ 172.6 (C-2), 163.1 (C-3a), 114.2 (C-3), 89.8 (C-4), 79.0 (C-7a), 70.7 (C-7), 62.4 (C-6); HRMS (ESI) *m/z* calcd for $C_7H_9O_5$ $[M+H]^{\cdot+}$ 173.0444, found 173.0436.

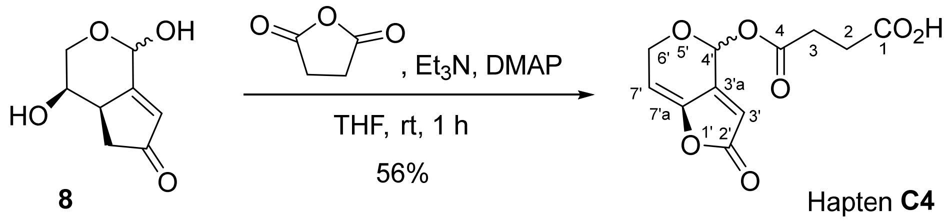

2.9. Preparation of (*R*)-4-oxo-4-((2-oxo-4,6-dihydro-2H-furo[3,2-c]pyran-4-yl)oxy) butanoic acid (Hapten C4). Compound **8** (28 mg, 0.162 mmol), succinic anhydride (35.6 mg, 0.356 mmol, 2.2 equiv) and DMAP (2.0 mg, 0.016 mmol, 0.1 equiv) were dissolved in anhydrous THF (0.45 mL) under nitrogen. Et_3N (100 µL, 0.712 mmol, 4.4 equiv) was then added dropwise and stirred for 1 hour at rt. The mixture was diluted with H_2O and acidified with 10% aqueous HCl to a pH of 4-5. The aqueous layer was then extracted with EtOAc and the combined organic layers were washed with brine, dried over anhydrous $MgSO_4$, and concentrated in vacuo to give hapten **C4** (24,7 mg, 56%) as an amorphous white solid. IR ν_{max} (cm^{-1}) 2967w, 2926w, 1862w, 1782s, 1754s, 1421m, 1240m, 1212m, 1230w, 1162m, 1113m, 1063m, 1048s, 921m, 910s, 812w; ^1H NMR (300 MHz, acetone-d_6) δ 7.04 (s, 1 H, H-3'), 6.19 (dq, *J* = 1.2, 0.6 Hz, 1H, H-4'), 6.14 (dt, *J* = 4.5, 2.4 Hz, 1H, H-7'), 4.73 (ddd, *J* = 17.6, 2.4, 1.2 Hz, 1H, H-6'), 4.52 (dd, *J* = 17.6, 4.5 Hz, 1H, H'-6'), 2.70-2.60 (m, 4H, H-2 and H-3); ^{13}C NMR (75 MHz, acetone-d_6) δ 173.5 (C-1), 173.2 (C-2'), 171.4 (C-4), 168.9 (C-3'a), 148.0 (C-7'a), 112.7 (C-3'), 108.3 (C-7'), 87.9 (C-4'), 61.2 (C-6'), 29.7 (C-3), 28.9 (C-2); HRMS (ESI) *m/z* calcd for $C_{11}H_{14}NO_7$ $[M+NH_4]^{+}$ 272.0765, found 272.0770.

3. Synthesis of hapten C6

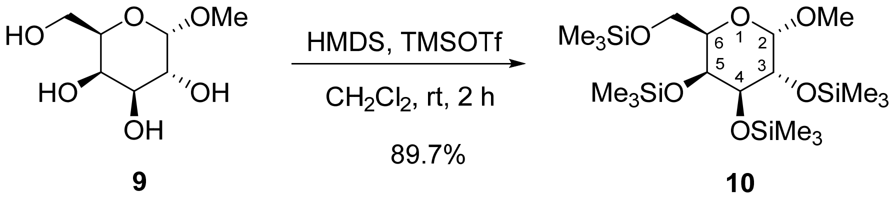

3.1. Preparation of (((2S,3*R*,4*S*,5*S*,6*R*)-2-methoxy-6-(((trimethylsilyl)oxy)methyl)tetra hydro-2H-pyran-3,4,5-triyl)*tris*(oxy))*tris*(trimethylsilane) (10). Methyl α-D-galactopyranoside (**9**) (1.487 g, 7.66 mmol) was dissolved in dry CH_2Cl_2 (15 mL) under nitrogen. The mixture was then cooled to 0 °C and hexamethyldisilazane (3.88 mL, 18.5 mmol, 2.4 equiv) and trimethylsilyl trifluoromethanesulfonate (150 µL, 183.7 mg, 0.837 mmol, 0.11 equiv) was added portion wise over 10 min. Upon complete addition, the mixture was warmed to rt and stirred for 2h, then diluted with hexane and washed with H_2O. The aqueous layer was extracted with EtOAc and the

combined organic layers were washed with brine, dried over anhydrous $MgSO_4$, and concentrated under reduced pressure to give **10** (3.314 g, 89.7%) as a clear oil. The product was pure enough by ^1H NMR to be used directly in the next step without further purification. $[\alpha]_D$= +73° (c 2.2, $CHCl_3$) {Lit.[6] $[\alpha]_D$= +81.5°}; IR ν_{max} (cm^{-1}) 2956w, 2897w, 1249m, 1102m, 1042w, 1002w, 967w, 923w, 873m, 832s, 747m, 685w; ^1H NMR (300 MHz, $CDCl_3$) δ 4.63 (d, *J* = 3.6 Hz, 1H, H-2), 3.92 (dd, *J* = 9.3, 3.6 Hz, 1H, H-3), 3.90 (m, 1H, H-5), 3.81 (dd, *J* = 9.6, 2.7 Hz, 1H, H-4), 3.72 (m, 1H, H-6), 3.64 (dd, *J* = 9.6, 6.9 Hz, 1H, H-1′), 3.59 (dd, *J* = 9.6, 6.3 Hz, 1H, H′-1′), 3.37 (s, 3H, MeO), 0.15, 0.14, 0.13 and 0.12 (each s, 9H each, 4xTMS); ^{13}C NMR (75 MHz, $CDCl_3$) δ 100.9 (C-2), 72.4 (C-5), 71.1 (2C, C-4 and C-6), 69.9 (C-3), 61.5 (C-1′), 55.4 (MeO), 0.8, 0.7, 0.5 and –0.4 (4xTMS); HRMS (ESI) *m/z* calcd for $C_{19}H_{46}NaO_6Si_4$ $[M+Na]^{\cdot+}$ 505.2264, found 505.2255.

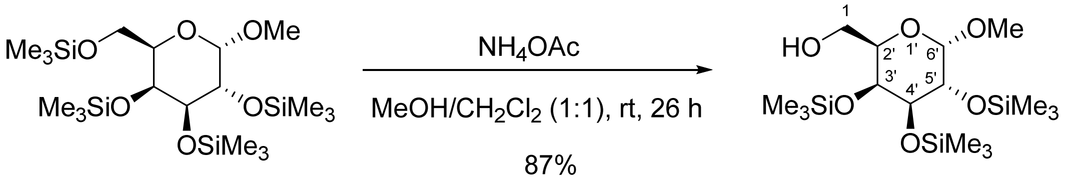

10 **11**

3.2. Preparation of ((2*R*,3*S*,4*S*,5*R*,6*S*)-6-methoxy-3,4,5-*tris*((trimethylsilyl)oxy)tetrahydro-2H-pyran-2-yl)methanol (11). Compound **10** (2.67 g, 5.53 mmol) and ammonium acetate (1.70 g, 22.06 mmol, 4 equiv) were dissolved in a 1:1 mixture of CH_2Cl_2/MeOH (92 mL) and stirred at rt for 25 hours.[7] The mixture was then concentrated under reduced pressure, dissolved in hexane/ether (1:1), washed with H_2O and brine, dried over anhydrous Na_2SO_4, and concentrated in vacuo to give alcohol **11** (1.97 g, 87%) as a thick clear oil with sufficient purity to be used in the next step without further purification. $[\alpha]_D$= +140° (c 0.2, $CHCl_3$); IR ν_{max} (cm^{-1}) 2956w, 2899w, 1249m, 1143m, 1102m, 1038w, 971w, 924w, 872m, 838s, 750m, 687w; ^1H NMR (300 MHz, $CDCl_3$) δ 4.66 (d, *J* = 3.5 Hz, 1H, H-6′), 3.91 (dd, *J* = 9.4, 3.5 Hz, 1H, H-5′), 3.80 (m, 4H, H-2′, H-3′, H-4′ and H-1), 3.61 (m, 1H, H′-1), 3.37 (s, 3H, MeO), 2.12 (d, *J* = 7.1 Hz, 1H, OH), 0.14, 0.13 and 0.13 (each s, 9H each, 3xTMS); ^{13}C NMR (75 MHz, $CDCl_3$) δ 100.9 (C-6′), 77.4 (C-3′), 73.8 (C-2′), 70.9 (C-4′), 69.7 (C-5′), 63.3 (C-1), 55.5 (MeO), 0.8, 0.7 and 0.45 (3xTMS); HRMS (ESI) *m/z* calcd for $C_{16}H_{38}NaO_6Si_3$ $[M+Na]^{\cdot+}$ 433.1868, found 433.1867.

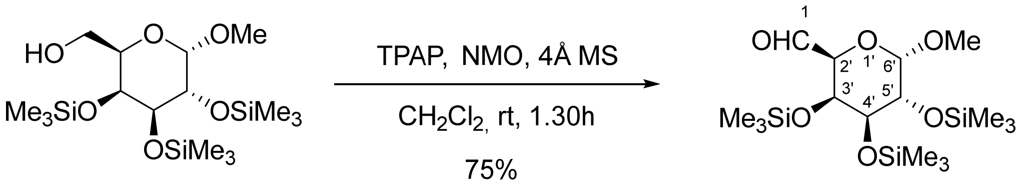

11 12

3.3. Preparation of (2*S*,3*S*,4*S*,5*R*,6*S*)-6-methoxy-3,4,5-*tris*((trimethylsilyl)oxy)tetrahydro-2H-pyran-2-carbaldehyde (12). A mixture of **11** (2.273 g, 5,33 mmol), *N*-methylmorpholine-*N*-oxide(NMO) (811 mg, 6.93 mmol), tetrabutylammonium perruthenate (TPAP) (187 mg, 0.53 mmol) and molecular sieves (4.404 g) in anhydrous CH_2Cl_2 (70 mL) was stirred at rt for 1.30h under nitrogen. After this time, isopropanol (3 mL) was added and the mixture was stirred for an additional 30 minutes. The residue left after evaporation of the solvents was chromatographed on silica gel, using a mixture of hexane-EtOAc-Et_3N (69:30:1) as eluent, to yield aldehyde **12** (1.697 g, 75%) as a yellowish oil. $[\alpha]_D$= +60° (c 0.2, $CHCl_3$); IR ν_{max} (cm^{-1}) 3412w, 2954w, 2923w, 2852w, 1781s, 1735s, 1671w, 1438w, 1345w, 1264w, 1170m, 1027m, 867w; ^1H NMR (300 MHz, $CDCl_3$) δ 9.60 (s, 1H, H-1), 4.79 (d, *J* = 3.5 Hz, 1H, H-6'), 4.25 (dd, *J* = 2.8, 1.7 Hz, 1H, H-3'), 4.08 (d, *J* = 1.7 Hz, 1H, H-2'), 3.99 (dd, *J* = 9.4, 3.4 Hz, 1H, H-5'), 3.83 (dd, *J* = 9.4, 2.8 Hz, 1H, H-4'), 3.43 (s, 3H, MeO), 0.16, 0.15 and 0.10 (each s, 9H each, 3xTMS); ^{13}C NMR (75 MHz, $CDCl_3$) δ 201.3 (C-1), 100.6 (C-6'), 75.9 (C-2'), 75.1 (C-4'), 69.7 (C-5'), 68.9 (C-3'), 55.5 (MeO), 0.6 (2xTMS), 0.4 (TMS); HRMS (ESI) *m/z* calcd for $C_{16}H_{36}NaO_6Si_3$ $[M+Na]^{+}$ 431.1712, found 431.1764.

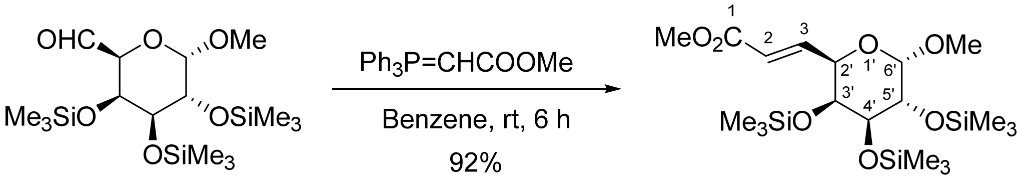

12 13

3.4. Preparation of (*E*)-methyl 3-((2*R*,3*S*,4*S*,5*R*,6*S*)-6-methoxy-3,4,5-*tris*((trimethylsilyl) oxy) tetrahydro-2H-pyran-2-yl)acrylate (13). Aldehyde **12** (1.92 g, 4.70 mmol) and methyl 2-(triphenylphosphoranylidene)acetate (1.93 g, 5.77 mmol, 1.23 equiv) were suspended in dry benzene under nitrogen. The mixture was stirred for 6 hours at rt, the solvent was eliminated at reduced pressure, and the residue obtained was purified by silica gel flash chromatography, using a mixture of hexane-EtOAc-Et_3N (79:20:1) as eluent, to afford **13** (1.96 g, 92%) as a yellowish oil, an approximately 4:1 mixture of *E/Z* isomers. IR ν_{max} (cm^{-1}) 2970w, 1737s, 1721s, 1439w, 1365m, 1250w, 1229m, 1145w, 1102w, 1044w, 899w, 841m; ^1H NMR (300 MHz, $CDCl_3$) (only signals of the predominant *E*-isomer are given) δ 6.84 (dd, *J* = 15.7, 4.3 Hz, 1H, H-3), 6.84

(dd, J = 15.7, 1.9 Hz, 1H, H-2), 4.70 (d, J = 3.5 Hz, 1H, H-6'), 4.39 (d, J = 4.3 Hz, 1H, H-2'), 3.95 (m, 1H, H-5'), 3.86 (m, 2H, H-3' and H-4'), 3.75 (s, 3H, MeO_2C), 3.37 (s, 3H, MeO), 0.16, 0.15 and 0.09 (each s, 9H each, 3xTMS); ^{13}C NMR (75 MHz, $CDCl_3$) (only signals of the predominant *E*-isomer are given) δ 166.8 (C-1), 145.3 (C-3), 121.7 (C-2), 101.0 (C-6'), 74.9 (C-4'), 70.8 (C-5'), 70.3 (C-2'), 69.4 (C-3'), 55.7 (MeO), 51.7 (*Me*O_2C), 0.7, 0.7 and 0.4 (3xTMS); HRMS (ESI) *m/z* calcd for $C_{19}H_{40}NaO_7Si_3$ $[M+Na]^{\cdot+}$ 487.1974, found 487.1969.

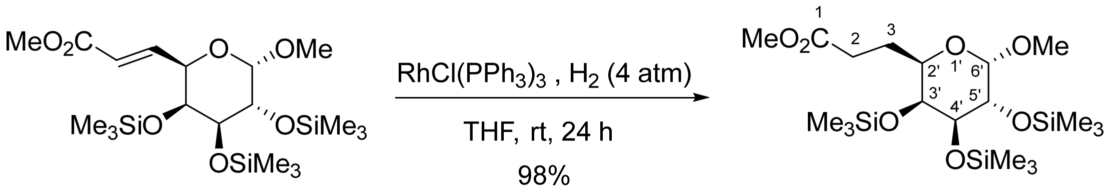

13 **14**

3.5. Preparation of methyl 3-((2*R*,3*S*,4*S*,5*R*,6*S*)-6-methoxy-3,4,5-*tris*((trimethylsilyl)oxy) tetrahydro-2H-pyran-2-yl)propanoate (14). Compound **13** (1.10 g, 2.37 mmol) and Wilkinson's catalyst (120 mg, 0.12 mmol, 0.05 equiv) were suspended in dry THF under nitrogen. The reactor was flushed with hydrogen several times, pressurized with hydrogen gas at 4 bar, and stirred at rt for 24 hours. The mixture was then diluted in diethyl ether, washed with H_2O and brine, dried over anhydrous $MgSO_4$, and concentrated in vacuo. The resulting mixture was purified by silica gel flash chromatography, using a mixture of hexane-EtOAc-Et_3N (79:20:1) as eluent, to afford **14** (1.081 g, 98%) as an oil. $[\alpha]_D$= +91° (c 1.9, $CHCl_3$); IR ν_{max} (cm^{-1}) 3056w, 2955w, 1737s, 1715s, 1359w, 1249m, 1143w, 1098m, 1042m, 896m, 876w; ^1H NMR (300 MHz, $CDCl_3$) δ 4.60 (d, J = 3.6 Hz, 1H, H-6'), 3.90 (dd, J = 9.6, 3.6 Hz, 1H, H-5'), 3.79 (dd, J = 9.6, 2.8 Hz, 1H, H-4'), 3.68 (m, 2H, H-2' and H-3'), 3.67 (s, 3H, MeO_2C), 3.32 (s, 3H, MeO), 2.44 (m, 2H, H-2), 1.98 (m, 1H, H-3), 1.68 (m, 1H, H'-3), 0.14, 0.13 and 0.12 (each s, 9H each, 3xTMS); ^{13}C NMR (75 MHz, $CDCl_3$) δ 174.1 (C-1), 100.8 (C-6'), 75.2 (C-2'), 71.1 (C-4'), 69.6 (C-5'), 69.4 (C-3'), 55.3 (MeO), 51.7 (*Me*O_2C), 30.3 (C-2), 26.6 (C-3), 0.9, 0.7 and 0.5 (3xTMS); HRMS (ESI) *m/z* calcd for $C_{19}H_{42}NaO_7Si_3$ $[M+Na]^{\cdot+}$ 489.2131, found 489.2126.

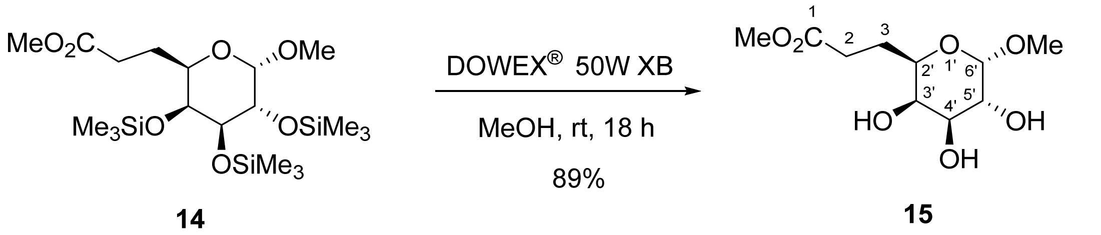

3.6. Preparation of methyl 3-((2*R*,3*R*,4*S*,5*R*,6*S*)-3,4,5-trihydroxy-6-methoxytetrahydro-2H-pyran-2-yl)propanoate (15). Compound **14** (1.031 g, 2.22 mmol) was dissolved in dry methanol (60 mL) under nitrogen. DOWEX® 50W XB resin (360 mg) was then added, and the mixture was stirred for 18 hours at rt. The resulting mixture was filtered through Celite and concentrated in vacuo to give triol **15** as a whitish solid (0.496 g, 89%) with sufficient purity to be used in the next step without further purification. Mp 131-133°C (MeOH); $[\alpha]_D$= +139° (c 1.9, $CHCl_3$); IR ν_{max} (cm^{-1}) 3539m, 3450s, 3312s, 3156m, 2956w, 2930w, 1746s, 1725s, 1628w, 1440w, 1370w, 1269w, 1195w, 1039m, 1041m, 974w, 782m; ^{1}H NMR (300 MHz, $CDCl_3$) δ 4.78 (d, *J* = 3.1 Hz, 1H, H-6'), 3.83 (m, 1H, H-5'), 3.79-3.72 (m, 3H, H-2', H-3' and H-4'), 3.69 (s, 3H, MeO_2C), 3.40 (s, 3H, MeO), 2.51 (m, 2H, H-2), 2.10 (m, 1H, H-3), 1.92 (m, 1H, H'-3); ^{13}C NMR (75 MHz, $CDCl_3$) δ 174.4 (C-1), 99.8 (C-6'), 71.2 (C-2'), 70.1 (C-5'), 69.1 (C-4'), 69.0 (C-3'), 55.3 (MeO), 51.8 (*Me*O_2C), 30.4 (C-2), 25.8 (C-3); HRMS (ESI) *m/z* calcd for $C_{10}H_{18}NaO_7$ $[M+Na]^{+}$ 273.0945, found 273.0946.

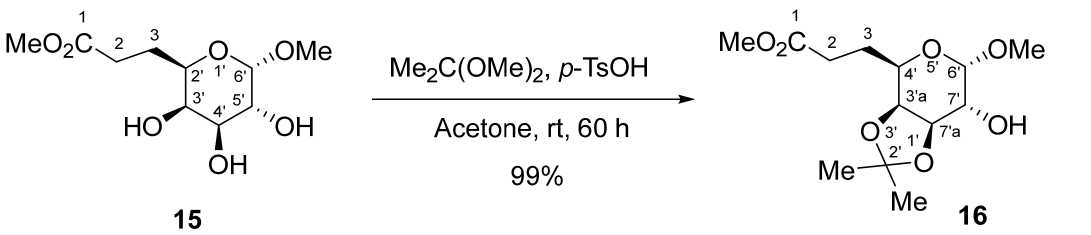

3.7. Preparation of methyl 3-((3a*S*,4*R*,6*S*,7*R*,7a*R*)-7-hydroxy-6-methoxy-2,2-dimethyltetrahydro-3aH-[1,3]dioxolo[4,5-c]pyran-4-yl)propanoate (16). A mixture of compound **15** (460 mg, 1.84 mmol) and *p*-toluenesulfonic acid (20 mg, 0.12 mmol) was dissolved in dry acetone (9 mL) under a nitrogen atmosphere. Then, 2,2-dimethoxypropane (274 µL, 0.233 mg, 2.23 mmol), 1.2 equiv) was added via syringe and the mixture stirred for 60 hours at rt. The reaction mixture was then quenched with a 5% aqueous solution of $NaHCO_3$ (2 mL) and diluted with CH_2Cl_2. The organic layer was washed with brine, dried over anhydrous $MgSO_4$, and concentrated under reduced pressure to give **16** (0.531 g, 99%) as a yellowish oil with sufficient purity to be used in the next step without further purification. $[\alpha]_D$= +115° (c 0.7, $CHCl_3$); IR ν_{max} (cm^{-1}) 3467s, 2986m, 2936m, 1735s, 1438w, 1370m, 1217w, 1195w, 1160m, 1063m, 1029m,

990w, 872m, 797m; ^{1}H NMR (300 MHz, $CDCl_3$) δ 4.71 (d, *J* = 3.9 Hz, 1H, H-6'), 4.19 (t, *J* = 6.3 Hz, 1H, H-7'a), 4.09 (dd, *J* = 6.0, 2.3 Hz, 1H, H-3'a), 3.95 (ddd, *J* = 9.4, 4.3, 2.2 Hz, 1H, H-4'), 3.78 (td, *J* = 6.6, 3.9 Hz, 1H, H-7'), 3.68 (s, 3H, MeO_2C), 3.41 (s, 3H, MeO), 2.51 (m, 2H, H-2), 2.27 (d, *J* = 6.7 Hz, 1H, OH), 2.07 (m, 2H, H-3), 1.50 and 1.34 (each s, 3H each, Me_2C); ^{13}C NMR (75 MHz, $CDCl_3$) δ 173.9 (C-1), 109.5 (C-2'), 98.7 (C-6'), 76.4 (C-4'), 74.9 (C-3'), 69.7 (C-5'), 69.5 (C-7'), 55.4 (MeO), 51.7 (MeO_2C), 30.4 (C-2), 27.9 and 26.1 (Me_2C), 26.1 (C-3); HRMS (ESI) *m/z* calcd for $C_{13}H_{22}NaO_7$ $[M+Na]^{+}$ 313.1258, found 313.1266.

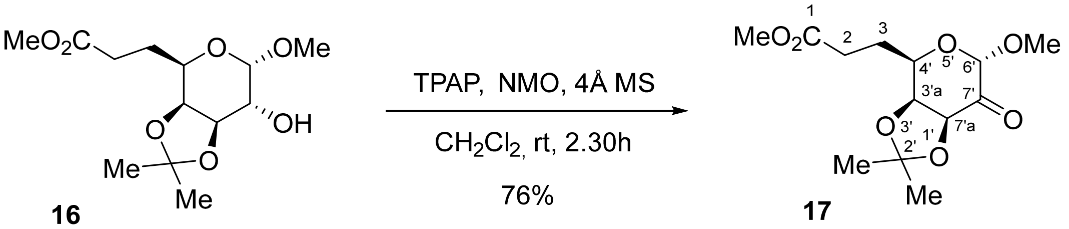

16 17

3.8. Preparation of methyl 3-((3a*S*,4*R*,6*S*,7a*S*)-6-methoxy-2,2-dimethyl-7-oxotetrahydro-3aH-[1,3]dioxolo[4,5-c]pyran-4-yl)propanoate (17). A mixture of **16** (552 mg, 1.90 mmol), NMO (285 mg, 1.22 mmol), (TPAP (74 mg, 0.21 mmol) and recently activated molecular sieves (400 mg) in anhydrous CH_2Cl_2 (27 mL) was stirred at rt for 2.30h under nitrogen. Isopropanol (2 mL) was then added and the mixture was stirred for an additional 30 minutes. The reaction mixture was concentrated at reduced pressure and the obtained residue was chromatographed on silica gel, using a 7:3 mixture of hexane-EtOAc as eluent, to yield ketone **17** (419 mg, 76%) as a yellowish oil. $[\alpha]_D$= +83° (c 0.7, $CHCl_3$); IR ν_{max} (cm^{-1}) 3001w, 2970w, 2942w, 1738s, 1440s, 1228m, 1217m, 1109w, 1078w, 870m, 785m; ^{1}H NMR (300 MHz, $CDCl_3$) δ 4.68 (s, 1H, H-6'), 4.62 (d, *J* = 5.6 Hz, 1H, H-7'a), 4.40 (dd, *J* = 5.6, 1.9 Hz, 1H, H-3'a), 4.30 (ddd, *J* = 9.5, 4.1, 1.9 Hz, 1H, H-4'), 3.70 (s, 3H, MeO_2C), 3.44 (s, 3H, MeO), 2.54 (m, 2H, H-2), 2.08 (m, 2H, H-3), 1.44 and 1.37 (each s, 3H each, Me_2C); ^{13}C NMR (75 MHz, $CDCl_3$) δ 199.4 (C-7'), 173.7 (C-1), 110.9 (C-2'), 100.6 (C-6'), 79.5 (C-3'a), 75.7 (C-7'a), 66.4 (C-4'), 55.6 (MeO), 51.8 (MeO_2C), 30.3 (C-2), 27.3 and 26.3 (Me_2C), 26.0 (C-3); HRMS (ESI) *m/z* calcd for $C_{13}H_{20}NaO_7$ $[M+Na]^{+}$ 311.1101, found 311.1144.

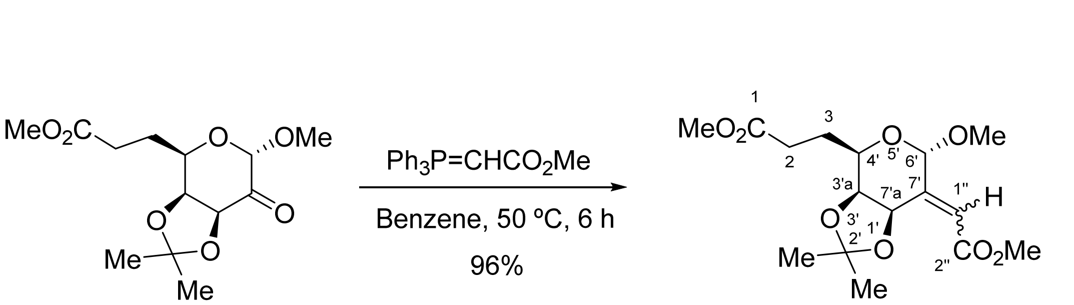

17 18

3.9. Preparation of methyl 3-((3a*S*,4*R*,6*S*,7a*R*)-6-methoxy-7-(2-methoxy-2-oxoethylidene)-2,2-dimethyltetrahydro-3aH-[1,3]dioxolo[4,5-c]pyran-4-yl)propanoate (18). Ketone **17** (400 mg, 1.39 mmol) and methyl 2-(triphenylphosphoranylidene)acetate (585 mg, 1.75 mmol, 1.25 equiv) were suspended in dry benzene (23 mL) under nitrogen. The mixture was stirred for 6 hours at 50 °C, cooled down to rt, and the solvent removed under vacuum. The obtained residue was purified by silica gel flash chromatography, using hexane-EtOAc mixtures from 9:1 to 1:1 as eluent, to afford unsaturated methyl ester **18** (459 mg, 96%) as a clear oil, a 4:1 mixture of *E/Z* stereoisomers. IR ν_{max} (cm^{-1}) 3003w, 2969w, 2951w, 1737s, 1726s, 1435m, 1365s, 1227m, 1216s, 1154w, 1090w, 1044w, 1009w; ^{1}H NMR (300 MHz, $CDCl_3$) (only signals of the major *E*-isomer are given) δ 6.35 (d, *J* = 1.6 Hz, 1H, H-1''), 6.05 (d, *J* = 7.6 Hz, 1H, H-7'a), 5.24 (d, *J* = 1.6 Hz, 1H, H-6'), 4.20 (dd, *J* = 7.6, 1.6 Hz, 1H, H-3'a), 3.74 (s, 3H, MeO_2C-2''), 3.67 (s, 3H, MeO_2C-1), 3.51 (m, 1H, H-4'), 3.46 (s, 3H, MeO), 2.49 (m, 2H, H-2), 2.01 (m, 1H, H-3), 1.86 (m, 1H, H'-3), 1.50 and 1.39 (each s, 3 H each, Me_2C); ^{13}C NMR (75 MHz, $CDCl_3$) (only signals of the major *E*-isomer are given) δ 173.7 (C-1), 165.9 (C-2''), 148.9 (C-7'), 123.8 (C-1''), 110.9 (C-2'), 98.0 (C-6'), 77.5 (C-3'a), 70.4 (C-4'), 69.4 (C-7'a), 55.6 (MeO), 52.0 (*Me*O_2C-2''), 51.9 (*Me*O_2C-1), 30.6 (C-2), 25.6 (C-3), 26.3 and 25.6 (Me_2C); HRMS (ESI) *m/z* calcd for $C_{16}H_{24}NaO_8$ $[M+Na]^{+}$ 367.1363, found 367.1388.

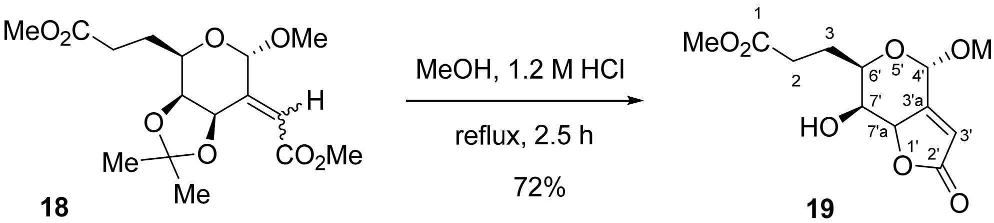

3.10. Preparation of methyl 3-((4*S*,6*R*,7*S*,7a*R*)-7-hydroxy-4-methoxy-2-oxo-4,6,7,7a-tetrahydro-2H-furo[3,2-c]pyran-6-yl)propanoate (19). Compound **18** (0.48 g, 1.39 mmol) and 2,6-di-*tert*-butyl-4-methylphenol (30.6 mg, 0.139 mmol, 0.1 equiv) were dissolved in dry MeOH (6 mL) under nitrogen. 334 µL of 1.2 M aqueous HCl (0.67 mmol, 0.5 equiv) was then added and the mixture was stirred at reflux for 2.5 hours. The mixture was then cooled to rt, diluted in H_2O, extracted with CH_2Cl_2, washed with brine, dried over anhydrous $MgSO_4$, and concentrated in vacuo. The residue obtained was purified by silica gel flash chromatography, using hexane-EtOAc mixtures from 8:2 to 6:4 as eluent, to afford **19** (0.27 g, 72%) as white crystals. Mp 88-89°C (MeOH); $[\alpha]_D$= +191° (c 0.5, $CHCl_3$); IR ν_{max} (cm^{-1}) 3459s, 2927m, 2850w, 1783w, 1732s, 1436w, 1346w, 1192w, 1144m, 1014m, 970w, 872m, 811w; ^{1}H NMR (300 MHz, $CDCl_3$) δ 6.00 (dd, *J* = 1.9, 0.7 Hz, 1H, H-3'), 5.47 (s, 1H, H-4'), 5.13 (dd, *J* = 3.9, 1.9 Hz, 1H, H-7'a), 4.21 (t, *J* = 4.2 Hz, 1H, H-

7’), 4.04 (dd, J = 9.7, 4.2 Hz, 1H, H-6’), 3.70 (s, 3H, MeO_2C-1), 3.47 (s, 3H, MeO), 2.53 (m, 2H, H-2), 2.14 (m, 1H, H-3), 2.00 (d, J = 5.1 Hz, 1H, HO), 1.93 (m, 1H, H’-3); ^{13}C NMR (75 MHz, $CDCl_3$) δ 173.7 and 172.5 (C-1 and C-2’), 159.4 (C-3’a), 115.7 (C-3’), 95.3 (C-4’), 79.2 (C-7’a), 71.5 (C-7’), 68.2 (C-6’), 55.3 (MeO), 51.9 (*Me*O_2C-1), 30.2 (C-2), 26.0 (C-3); HRMS (ESI) *m/z* calcd for $C_{12}H_{16}NaO_7$ $[M+Na]^{·+}$ 273.0969, found 273.0970.

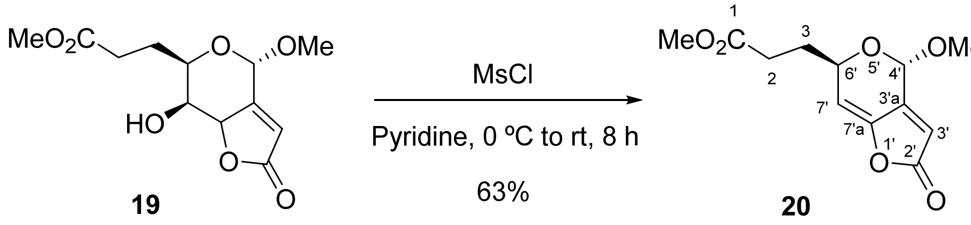

3.11. Preparation of methyl 3-((4*S*,6*R*)-4-methoxy-2-oxo-4,6-dihydro-2H-furo[3,2-c]pyran-6-yl)propanoate (20). Mesyl chloride (262 µL, 3.37 mmol, 3.5 equiv) was dropwise added to a solution of alcohol **19** (260 mg, 0.964 mmol) in anhydrous pyridine (5 mL) at 0 °C under nitrogen. The mixture was allowed to warm to rt and stirred for 8 hours, then diluted with CH_2Cl_2, washed successively with water, 1 M aqueous solution of $KHSO_4$, and brine, dried over anhydrous $MgSO_4$, and concentrated in vacuo. The residue obtained was purified by silica gel flash chromatography, using hexane-EtOAc mixtures from 7:3 to 6:4 as eluent, to afford the α,β,γ,δ-unsaturated lactone **20** (152 mg, 63%) as a white solid. Mp 76-77°C (Et_2O); $[\alpha]_D$= +109° (c 0.5, $CHCl_3$); IR ν_{max} (cm^{-1}) 2953w, 2928w, 1782s, 1733s, 1672w, 1438w, 1355m, 1260w, 1169s, 1072w, 1032w, 973w, 864w; ^1H NMR (300 MHz, $CDCl_3$) δ 5.97 (ddd, J = 1.9, 1.2, 0.5 Hz, 1H, H-3’), 5.78 (t, J = 2.0 Hz, 1H, H-7’), 5.62 (s, 1H, H-4’), 4.64 (ddt, J = 7.8, 3.8, 1.6 Hz, 1H, H-6’), 3.69 (s, 3H, MeO_2C-1), 3.52 (s, 3H, MeO), 2.52 (dd, J = 10.8, 4.7 Hz, 2H, H-2), 2.06 (m, 2H, H-3); ^{13}C NMR (75 MHz, $CDCl_3$) δ 173.5 (C-1), 168.7 (C-2’), 148.5 (C-3’a), 147.1 (C-7’a), 111.8 (C-3’), 110.4 (C-7’), 94.2 (C-4’), 66.8 (C-6’), 56.2 (MeO), 51.9 (*Me*O_2C-1), 30.1 (C-2), 29.5 (C-3); HRMS (ESI) *m/z* calcd for $C_{13}H_{18}NaO_7$ $[M+Na+CH_3OH]^{·+}$ 309.0945, found 309.0963.

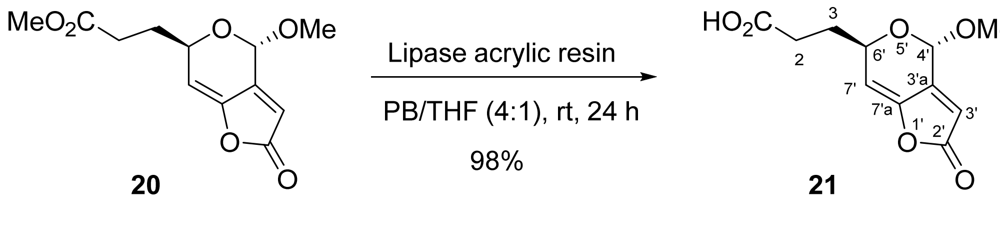

3.12. Preparation of methyl 3-((4*S*,6*R*)-4-methoxy-2-oxo-4,6-dihydro-2H-furo[3,2-c]pyran-6-yl)propanoate (21). Methyl ester **20** (150 mg, 0.590 mmol) was dissolved in 20 mL of

a 4:1 mixture of 100 mM sodium phosphate buffer (pH 7.4) and THF. Then, 300 mg of lipase acrylic resin from *Candida antarctica* was added and the mixture was smoothly stirred for 24 hours at rt. After filtering the enzyme, the filtrate was diluted with water, acidified to pH ≈ 4 with 1 M HCl, and extracted with EtOAc. The combined organic layers were washed with brine, dried over anhydrous $MgSO_4$, and concentrated in vacuo. The resulting residue was purified by silica gel flash chromatography, using $CHCl_3$-MeOH (98:2) as eluent, to afford acid **21** (139 mg, 98%) as an amorphous white solid. $[\alpha]_D$= +108° (c 0.6, $CHCl_3$); IR ν_{max} (cm^{-1}) 3109w, 3095w, 2836w, 1785s, 1703s, 1673m, 1623w, 1406m, 1349w, 1259m, 1907m, 1069s, 1027s, 961s, 859s, 839s, 806m; ^1H NMR (300 MHz, $CDCl_3$) δ 5.99 (m, 1H, H-3'), 5.78 (t, *J* = 2.0 Hz, 1H, H-7'), 5.63 (s, 1H, H-4'), 4.67 (ddt, *J* = 8.3, 3.7, 1.7 Hz, 1H, H-6'), 3.53 (s, 3H, MeO), 2.58 (dd, *J* = 10.8, 4.5 Hz, 2H, H-2), 2.09 (m, 2H, H-3); ^{13}C NMR (75 MHz, $CDCl_3$) δ 178.9 (C-1), 168.8 (C-2'), 148.4 (C-3'a), 147.2 (C-7'a), 111.9 (C-3'), 110.2 (C-7'), 94.3 (C-4'), 66.8 (C-6'), 56.2 (MeO), 29.9 (C-2), 29.5 (C-3); HRMS (ESI) *m/z* calcd for $C_{12}H_{14}NaO_6$ $[M+Na]^{\cdot+}$ 263.0526, found 263.0518.

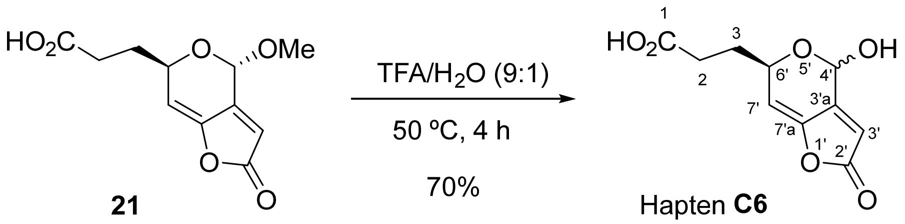

3.13. Preparation of 3-((4*S*,6*R*)-4-hydroxy-2-oxo-4,6-dihydro-2H-furo[3,2-c]pyran-6-yl)propanoic acid (hapten C6). Compound **21** (20 mg, 0.083 mmol) was dissolved in 1 mL of a 9:1 mixture of trifluoroacetic acid and H_2O and stirred at 50 °C for 4 hours. The mixture was then diluted with water and extracted with EtOAc. The organic layer was washed with brine, dried over anhydrous $MgSO_4$, and concentrated under reduced pressure. The residue obtained was purified by silica gel flash chromatography, using $CHCl_3$-MeOH (95:5) containing a 0.1% of HCO_2H as eluent, to afford hapten **C6** (13.2 mg, 70%) as a film. From the ^1H NMR data, this product was determined to be an approximately 2:1 mixture of epimers at hemiacetalic carbon C-4', in equilibrium with a small percentage of the open hydroxy-aldehyde form. IR ν_{max} (cm^{-1}) 3117w, 2926w, 2854w, 1779s, 1747s, 1707s, 1673m, 1624w, 1408m, 1171m, 861m; ^1H NMR (300 MHz, acetone-d_6) [signals of major α-OH epimer/signals of minor β-OH-epimer] δ 6.30/6.67 (br d, *J* = 5.5/7.0 Hz, 1H, OH), 6.17/5.91 (br d, *J* = 3.8/5.2 Hz, 1H, H-4'), 6.11/6.06 (ddd, *J* = 1.8, 1.1, 0.5/2.0, 1.4, 1.0 Hz, 1H, H-3'), 6.02/6.02 (t, *J* = 2.1 Hz, 1H, H-7'), 4.83/4.61 (m, 1H, H-6'), 2.51/2.47 (t, *J* = 7.5/7.3 Hz, 2H, H-2), 2.13-1.86 (m overlapped with solvent signal, 2H, H-3); ^{13}C NMR (75 MHz, $CDCl_3$) [signals of major α-OH epimer/signals of minor β-OH-epimer] δ 174.8/174.8 (C-1),

169.5/169.4 (C-2'), 152.1/155.1 (C-3'a), 147.8/149.1 (C-7'a), 111.7/112.1 (C-7'), 111.1/110.5 (C-3'), 88.4/91.5 (C-4'), 67.3/72.5 (C-6'), 30.8/31.5 (C-2), 29.8/29.8 (C-3); HRMS (ESI) *m/z* calcd for $C_{10}H_{10}NaO_6$ $[M+Na]^{·+}$ 249.0370, found 249.0365.

4. Hapten activation with *N*-hydroxysuccinimide (NHS) by carbodiimide-mediated chemistry

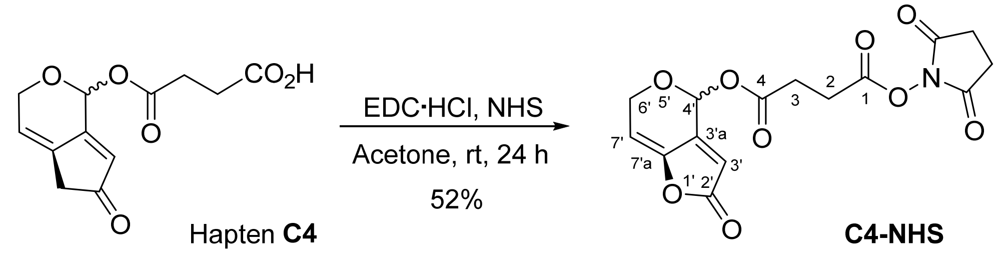

4.1. Preparation of 2,5-dioxopyrrolidin-1-yl (2-oxo-2,6-dihydro-4H-furo[3,2-c]pyran-4-yl) succinate (C4–NHS). A solution of hapten **C4** (13.3 mg, 0.05 mmol), NHS (13.4 mg, 0.116 mmol, 2.3 equiv) and *N*-(3-Dimethylaminopropyl)-*N'*-ethylcarbodiimide hydrochloride (EDC·HCl; 25.0 mg, 0.130 mmol, 2.6 equiv) in anhydrous acetone was stirred for 24 hours under nitrogen atmosphere. Upon completion, the reaction mixture was diluted with $CHCl_3$, washed with water and brine, and dried over anhydrous $MgSO_4$. After solvent evaporation, the residue was chromatographed through a small column of silica gel, employing $CHCl_3$ as eluent, to obtain the *N*-hydroxysuccinimidyl ester **C4–NHS** (8.4 mg, 52%) as a clear oil. ^{1}H NMR (300 MHz, $CDCl_3$) δ 7.02 (d, *J* = 0.5 Hz, 1H, H-4'), 6.06 (dt, *J* = 1.3, 0.7 Hz, 1H, H-3'), 5.95 (dt, *J* = 4.5, 2.2 Hz, 1H, H-7'), 4.72 (ddd, *J* = 17.5, 2.5, 1.3 Hz, 1H, H-6'), 4.51 (dd, *J* = 17.5, 4.6 Hz, 1H, H'-6'), 2.98 (m, 2H, H-2), 2.84 (s, 4H, $CO(CH_2)_2CO$), 2.81 (m, 2H, H-3).

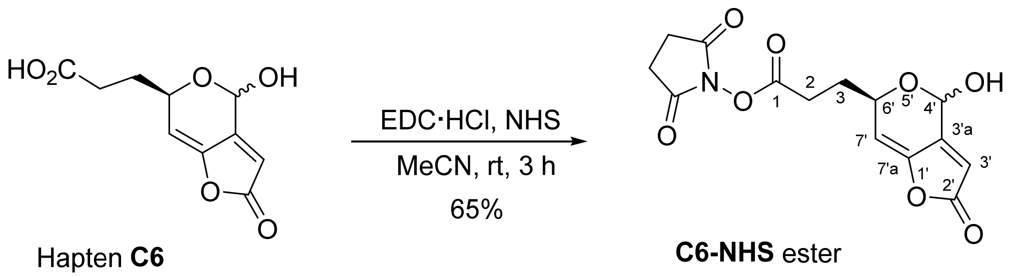

4.2. Preparation of 2,5-dioxopyrrolidin-1-yl 3-((6*R*)-4-hydroxy-2-oxo-2,6-dihydro-4H-furo[3,2-c]pyran-6-yl)propanoate (C6–NHS). A solution of hapten **C6** (14.4 mg, 0.062 mmol), NHS (7.5 mg, 0.065 mmol, 1.05 equiv) and EDC·HCl (12.4 mg, 0.065 mmol, 1.05 equiv) in anhydrous CH_3CN was stirred for 3 hours under nitrogen atmosphere. Upon completion, the

reaction mixture was diluted with CH_2Cl_2, washed with water and brine, and dried over anhydrous $MgSO_4$. Evaporation of the solvent under reduced pressure, followed by filtration through a short pad of silica gel, using $CHCl_3$-MeOH (95:5) as eluent, afforded the corresponding *N*-hydroxysuccinimidyl ester, **C6–NHS** (12.9 mg, 65%), as a light brownish solid, also a mixture of epimers at C-4'. ^{1}H NMR (300 MHz, $CDCl_3$) [signals of major α-OH epimer/signals of minor β-OH-epimer] 6.12/5.83 (br s/t, *J* = 1.2 Hz, 1H, H-4'), 6.07/6.02 (dt, *J* = 2.2, 1.2/1.8, 0.8 Hz, 1H, H-3'), 5.79/5.76 (t, *J* = 2.1 Hz, 1H, H-7'), 4.85/4.65 (dddd, *J* = 9.9, 3.7, 2.1, 1.1/7.0, 4.7, 2.3, 1.0 Hz, 1H, H-6'), 2.86 (s, 4H, $CO(CH_2)_2CO$), 2.65–2.85 (m, 2H, H-2), 2.34—2.05 (m, 2H, H-3).

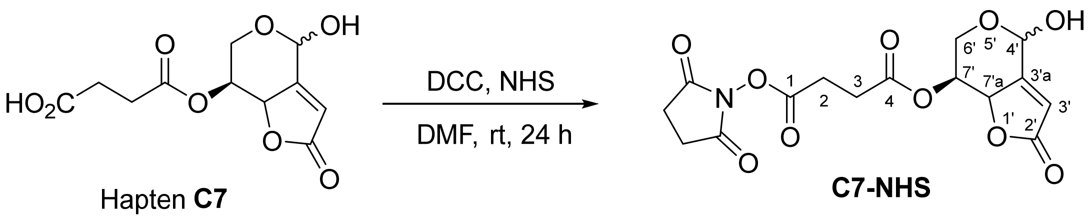

4.3. Preparation of 2,5-dioxopyrrolidin-1-yl ((4S,7S,7aR)-4-hydroxy-2-oxo-2,6,7,7a-tetrahydro-4H-furo[3,2-c]pyran-7-yl) succinate (C7–NHS). A solution of hapten **C7** (2.4 mg, 9 µmol), NHS (1.0 mg, 9 µmol, 1.0 equiv) and dicyclohexylcarbodiimide (DCC; 2.2 mg, 9 µmol, 1.0 equiv) in anhydrous DMF was stirred for 24 hours at rt under nitrogen. Upon completion, the reaction mixture was centrifuged to separate the activated hapten **C7–NHS** from the formed dicyclohexylurea. The collected supernatant was then directly used for the preparation of hapten-protein conjugates.

5. References

1. Perrin, D. D. & Armarego, W. L .F. in "Purification of Laboratory Chemicals", 4th ed.; Butterworth Heinemann Press: Oxford, 1996.
2. Smith, F. & Van Cleve, J. W. A note on the synthesis of glycosides. *J. Am. Chem. Soc.* **77**, 3159-3160 (1955).
3. Shing, T. K. M, Leung, Y. C. & Yeung, K. W. Catalytic asymmetric epoxidation of alkenes with arabinose-derived uloses. *Tetrahedron* **59**, 2159–2168 (2003).
4. Bennett, M., Gill, G. B., Pattenden, G., Shuker, A. J. & Stapleton, A. Ylidenebutenolide mycotoxins. Concise syntheses of patulin and neopatulin from carbohydrate precursors. *J. Chem. Soc. Perkin Trans.* **1**, 929–937 (1991).
5. Gill, G. B., Pattenden, G. & Stapleton, A. A concise synthesis of patulin from arabinose. *Tetrahedron Lett.* **29**, 2875–2878 (1998).
6. Joseph, A. A. *et al.* TMSOTf-catalyzed silylation: streamlined regioselective one-pot protection and acetylation of carbohydrates. *Eur. J. Org. Chem.* 744–753 (2012).
7. Jin, L. *et al.* Novel eco-friendly solution for the regioselective acetylation of per-O-TMS carbohydrates. *Carbohydr. Res.* **495**, 108074 (2020).

12. Copies of ^{1}H NMR spectra of haptens, adducts and *N*-hydroxysuccinimidyl esters

^{1}H NMR spectrum (300 MHz) of hapten **C4** in acetone d_6

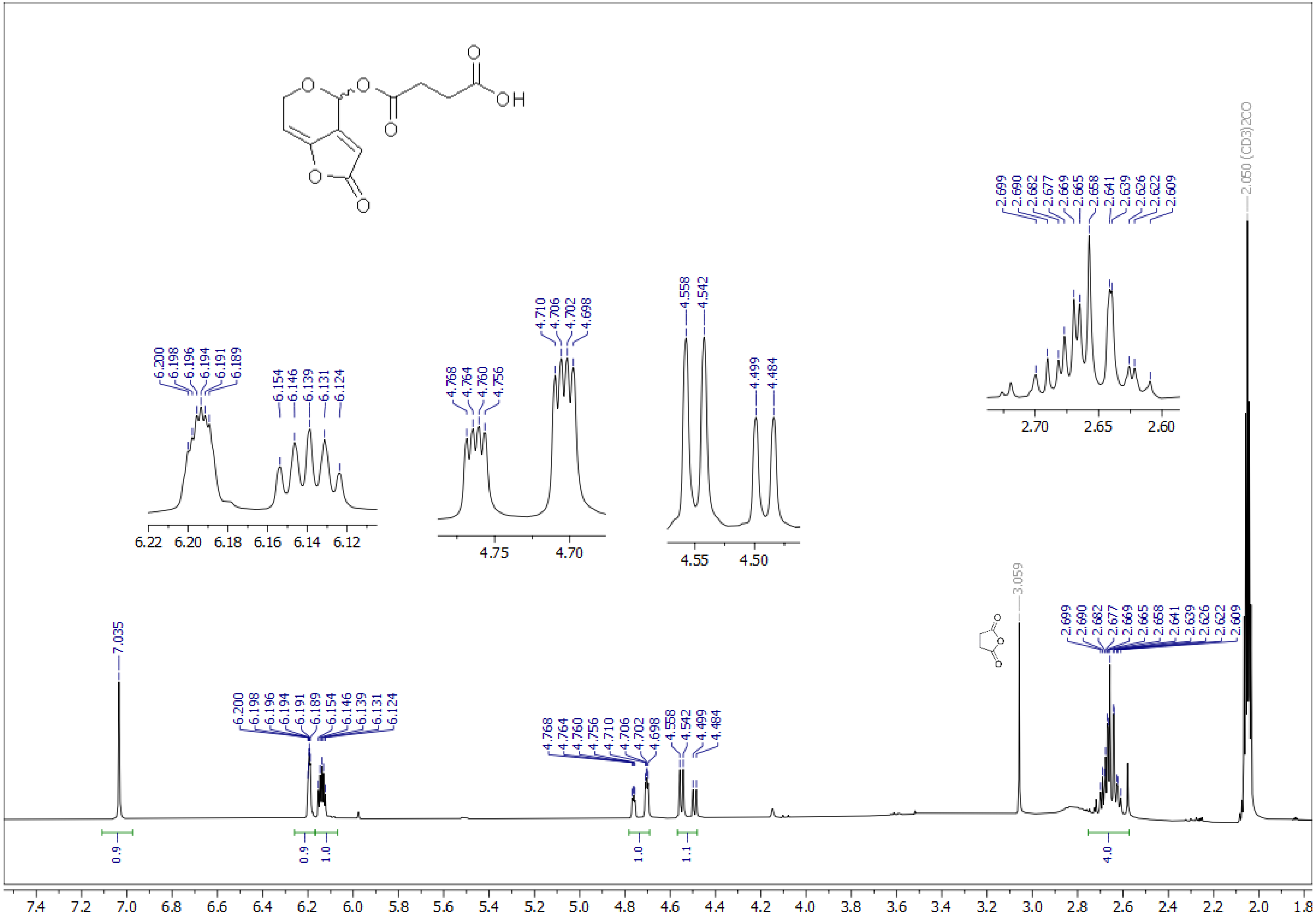

^{1}H NMR spectrum (300 MHz) of **C4–NHS** ester in $CDCl_3$

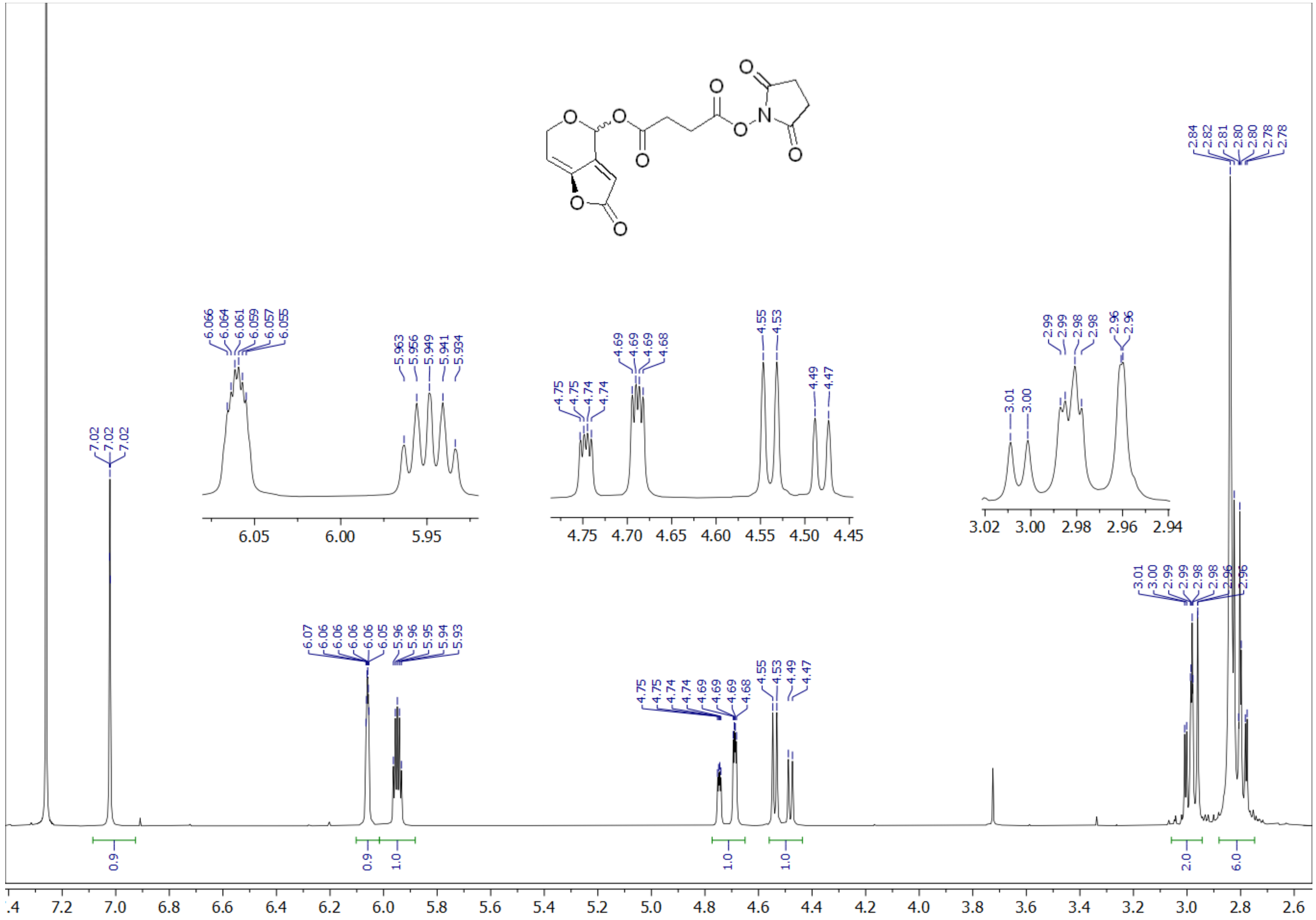

^{1}H NMR spectrum (300 MHz) of hapten **C6** in acetone d_6

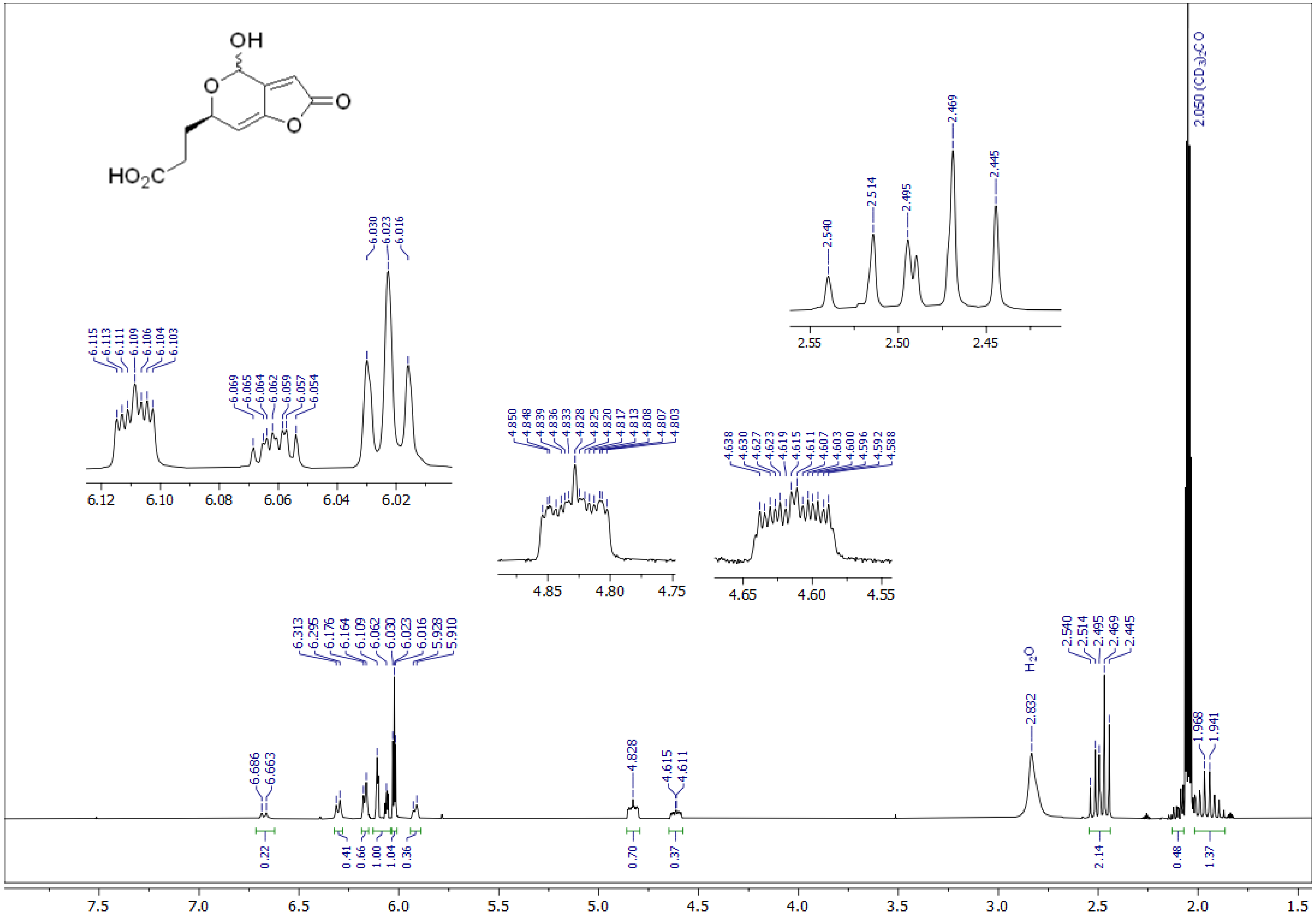

^{1}H NMR spectrum (300 MHz) of **C6–NHS** ester in $CDCl_3$

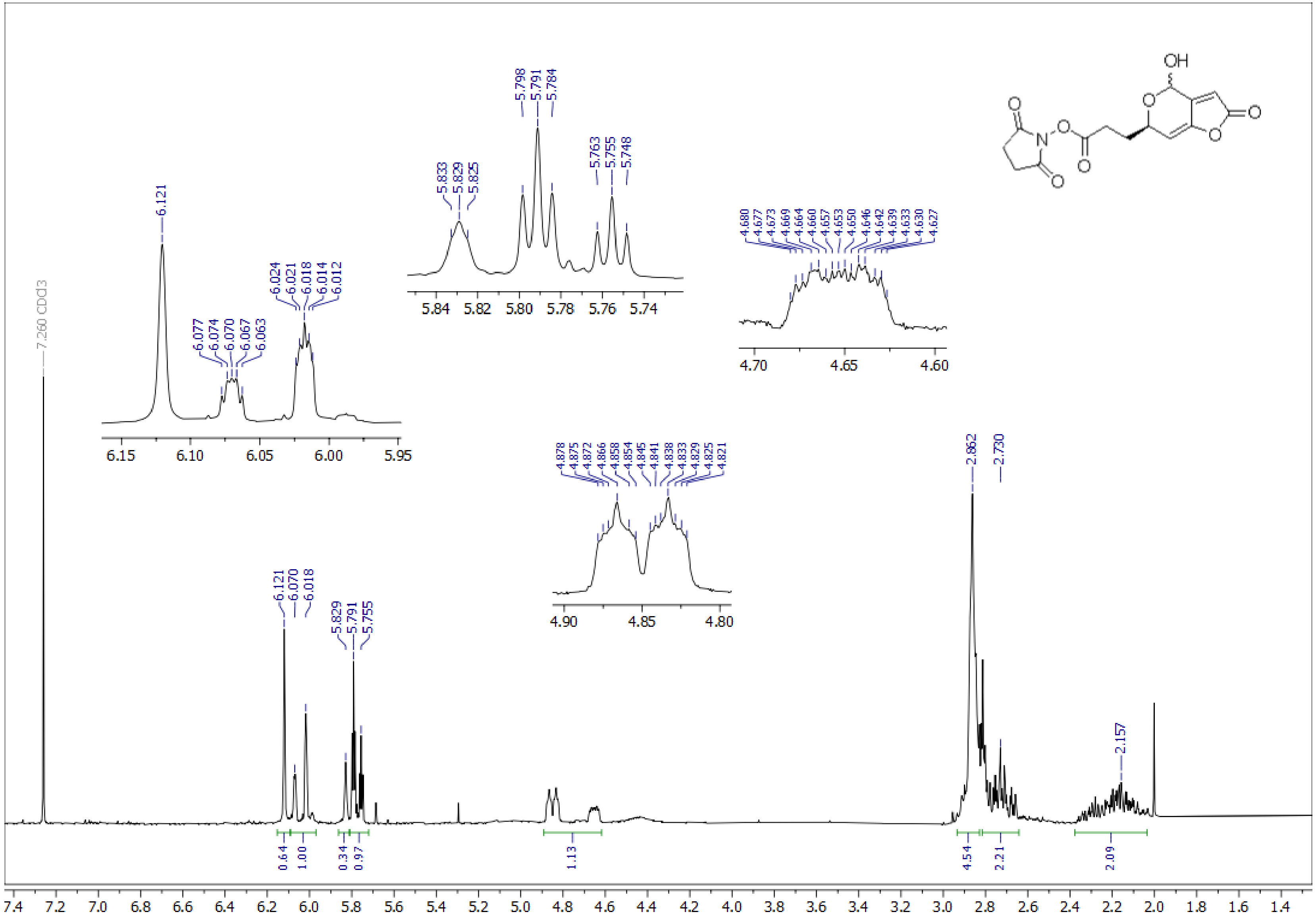

^{1}H NMR spectrum (300 MHz) of hapten **C7** in acetone d_6

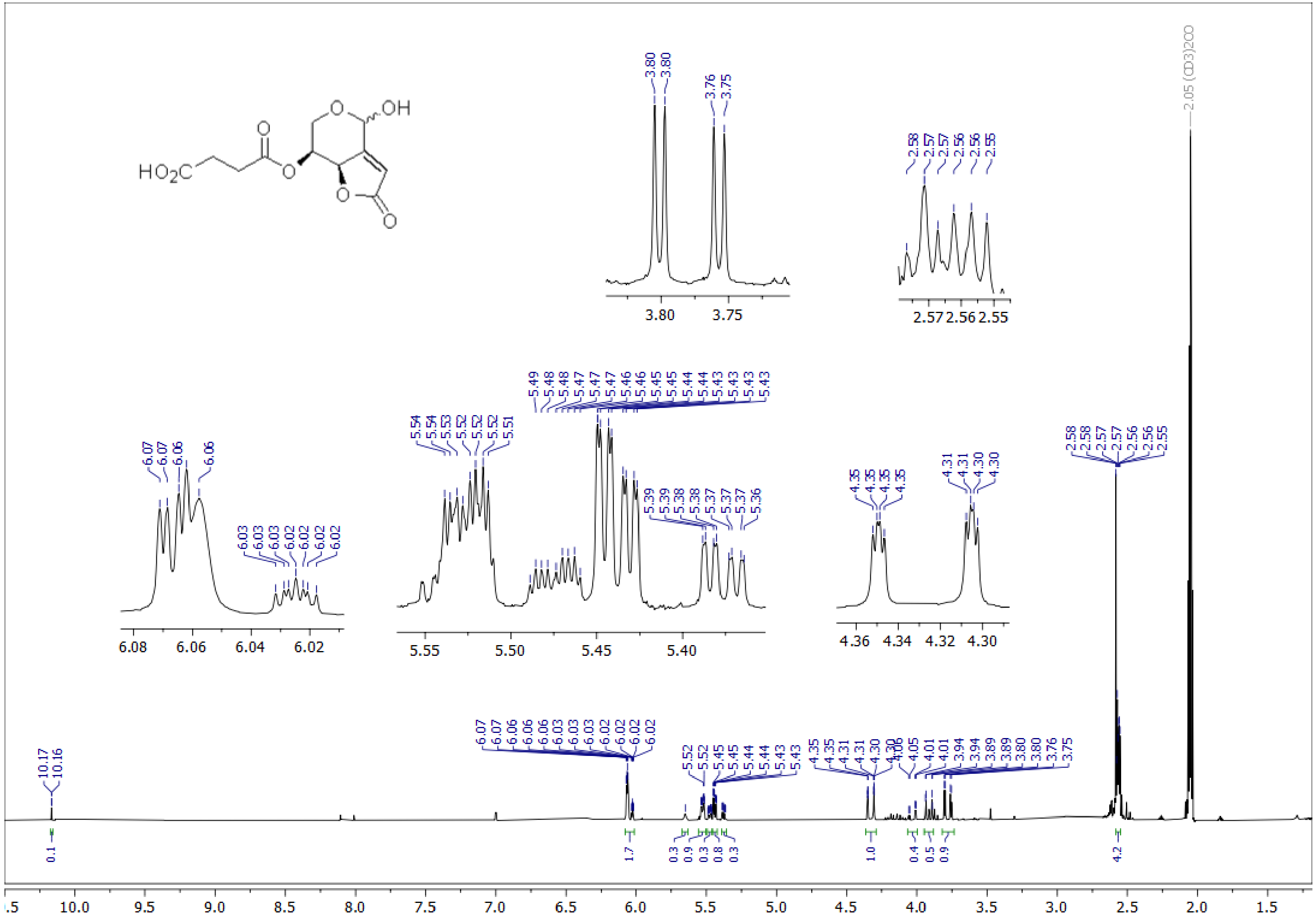

^{1}H NMR spectrum (500 MHz) of adduct **V** in acetone d_6

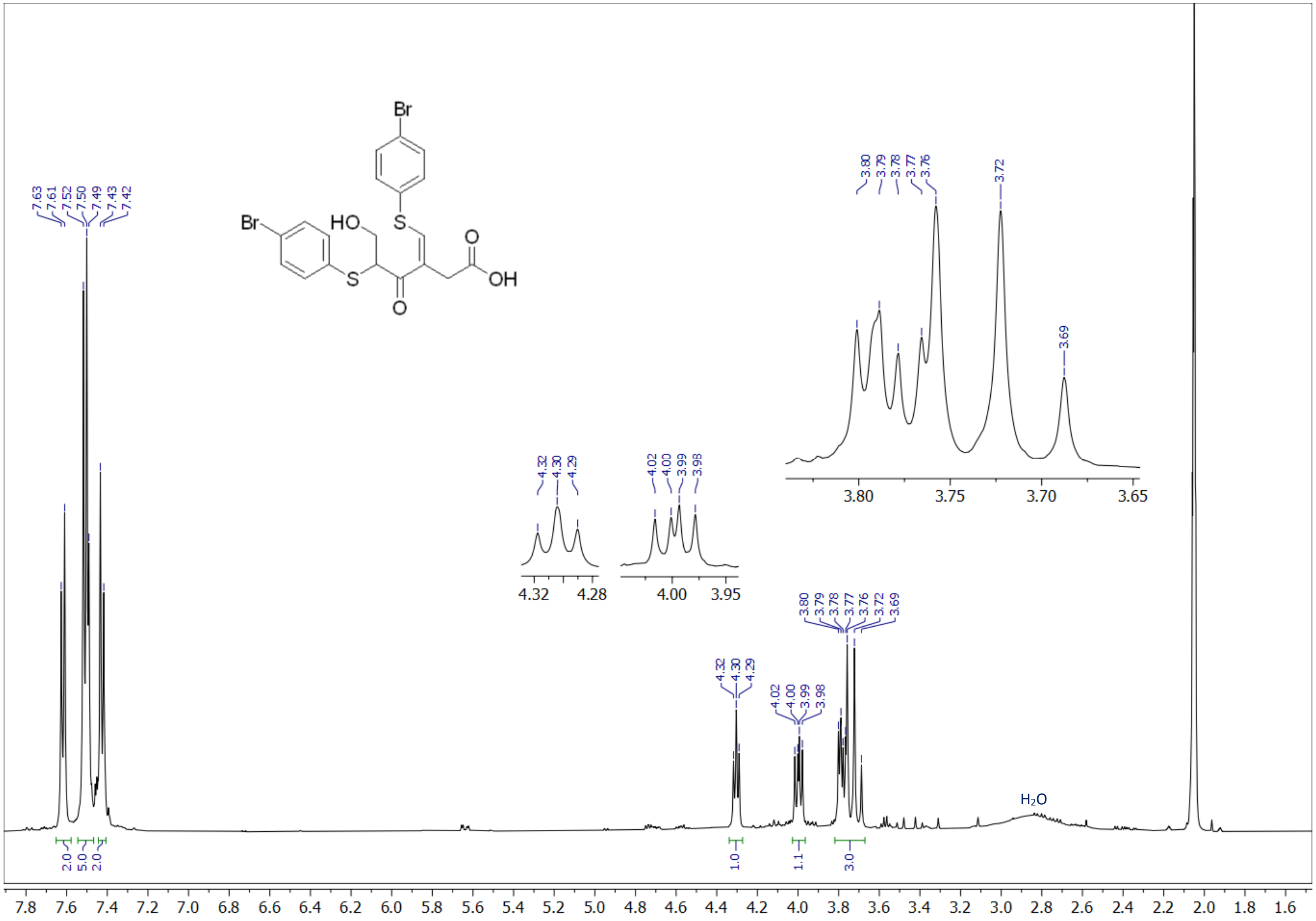

^{1}H NMR spectrum (300 MHz) of adduct **V–NHS** ester in $CDCl_3$

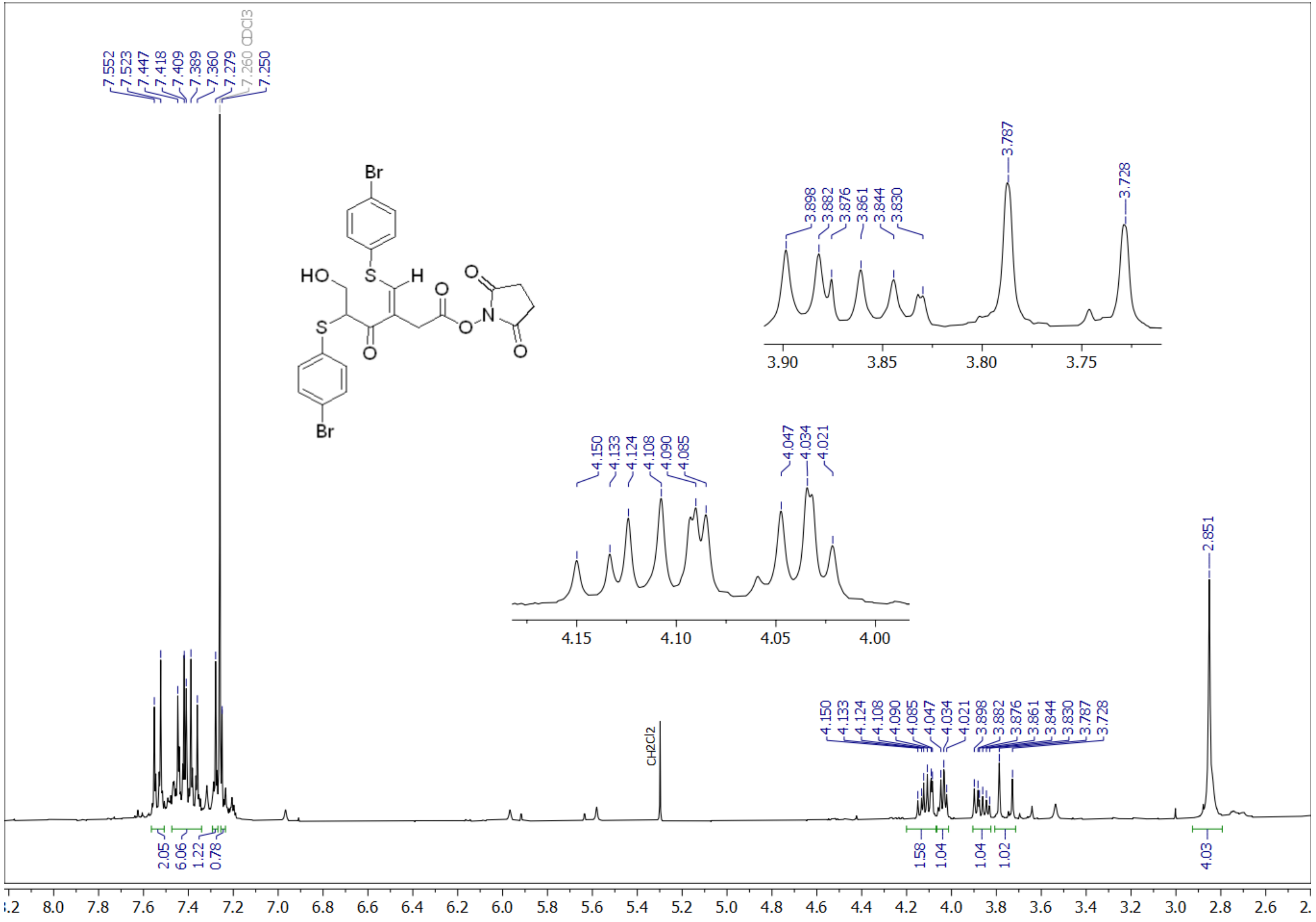

^{1}H NMR spectrum (300 MHz) of hapten **V4** in acetone d_6

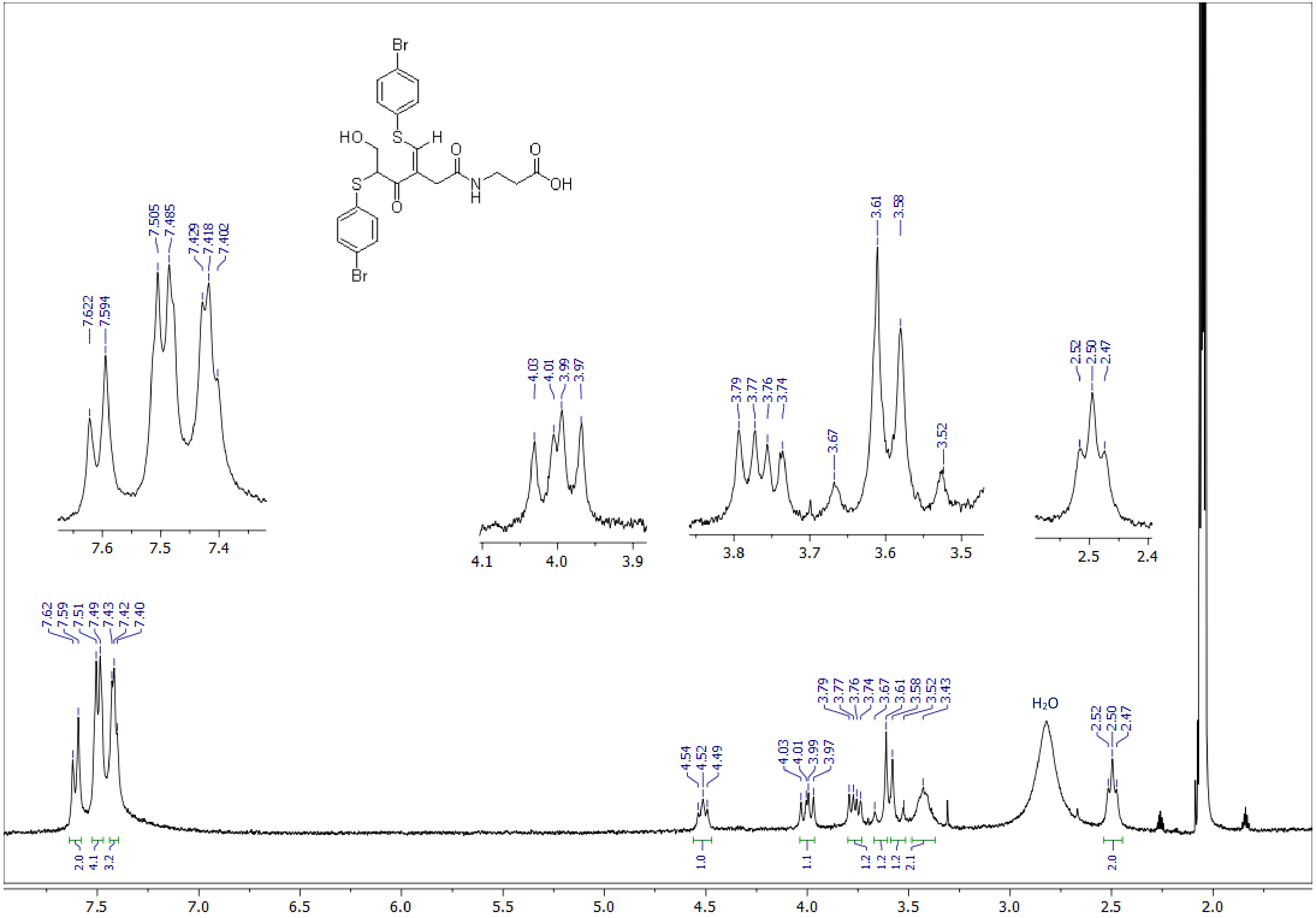

^{1}H NMR spectrum (300 MHz) of **V4–NHS** ester in acetone d_6

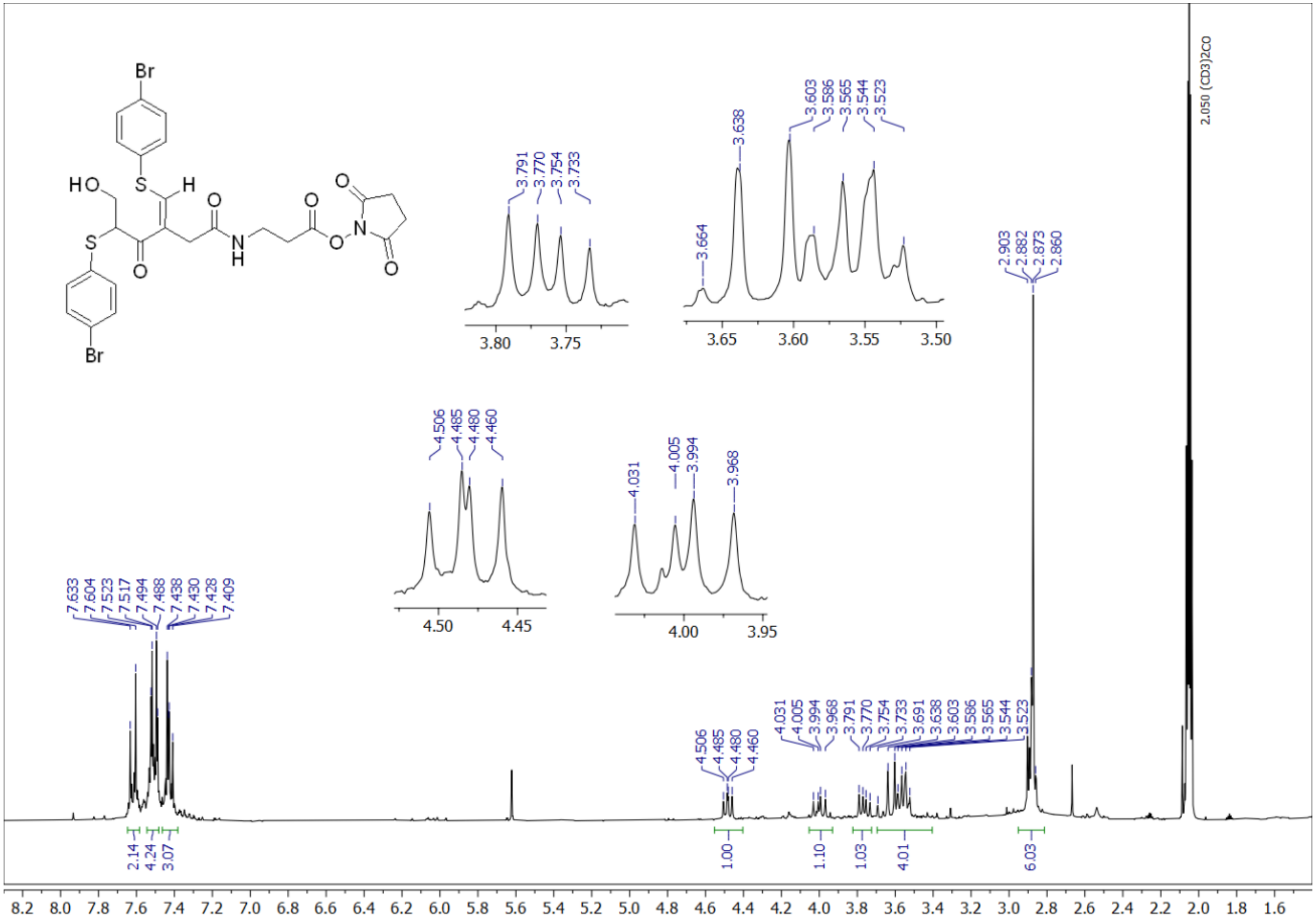

^{1}H NMR spectrum (300 MHz) of adduct **I** in $CDCl_3$

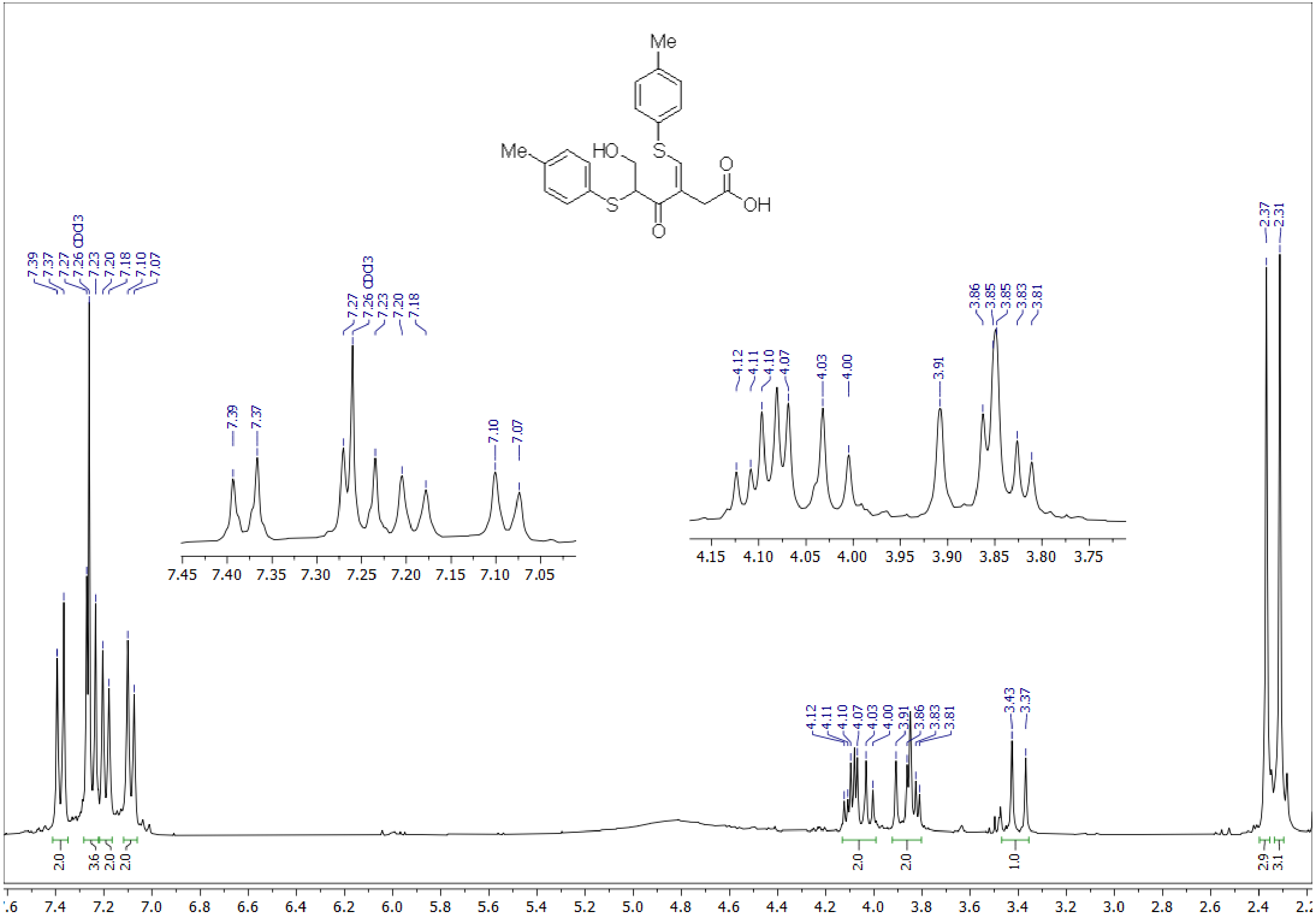

^{1}H NMR spectrum (300 MHz) of adduct **II** in $CDCl_3$

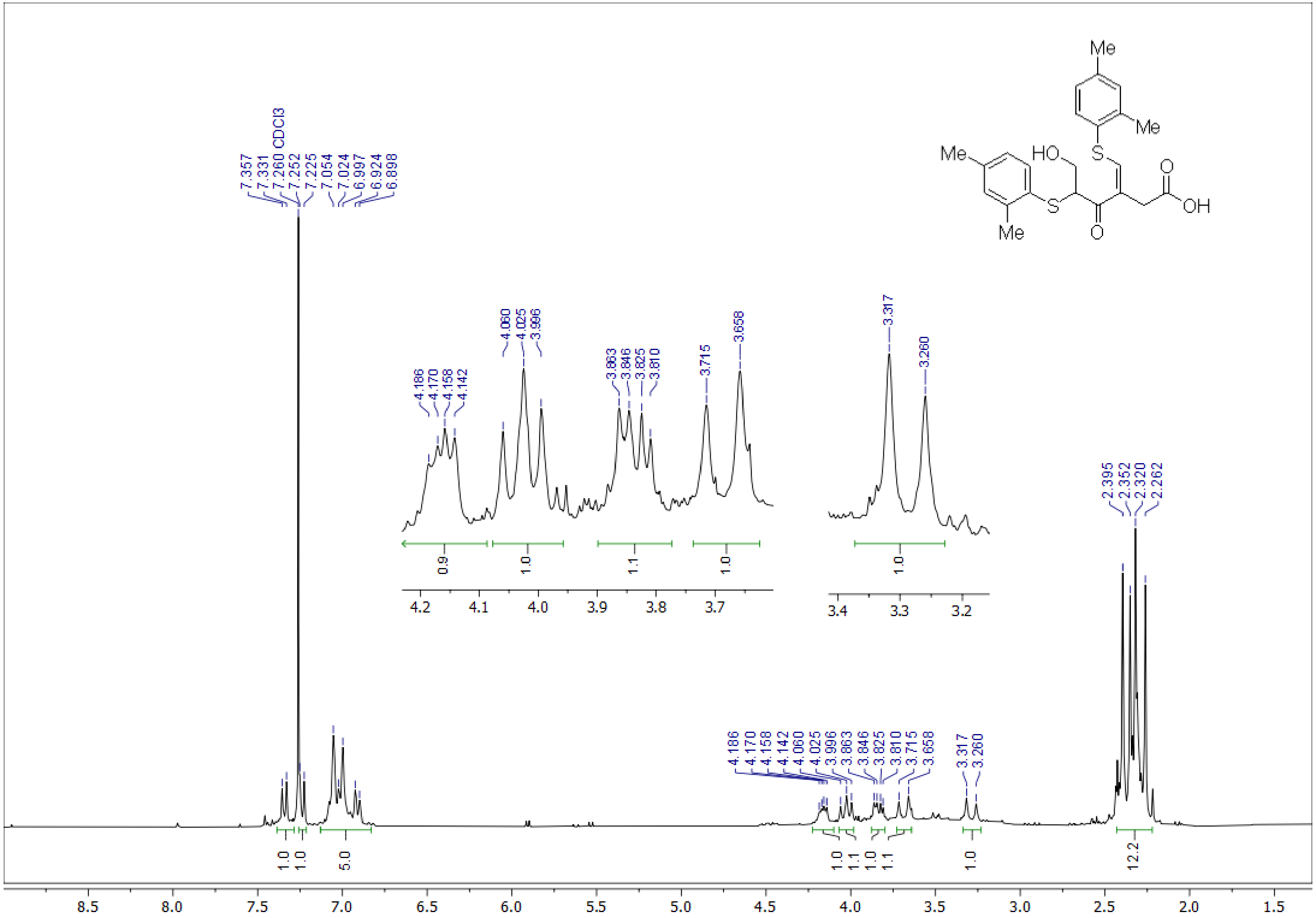

^{1}H NMR spectrum (300 MHz) of adduct **III** (mixture of *E/Z* isomers) in acetone d_6

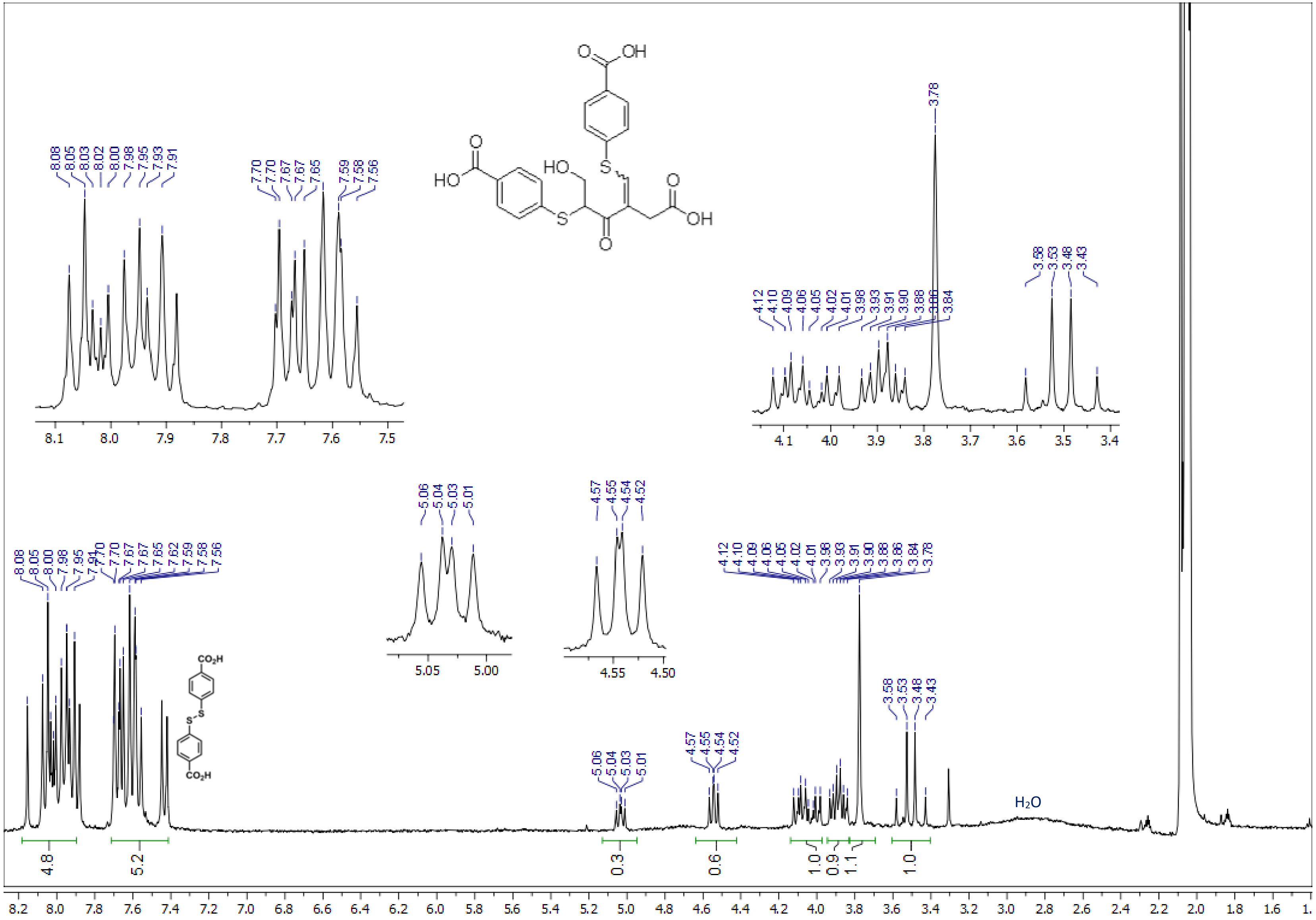

^1H NMR spectrum (500 MHz) of adduct **IV** in acetone d_6

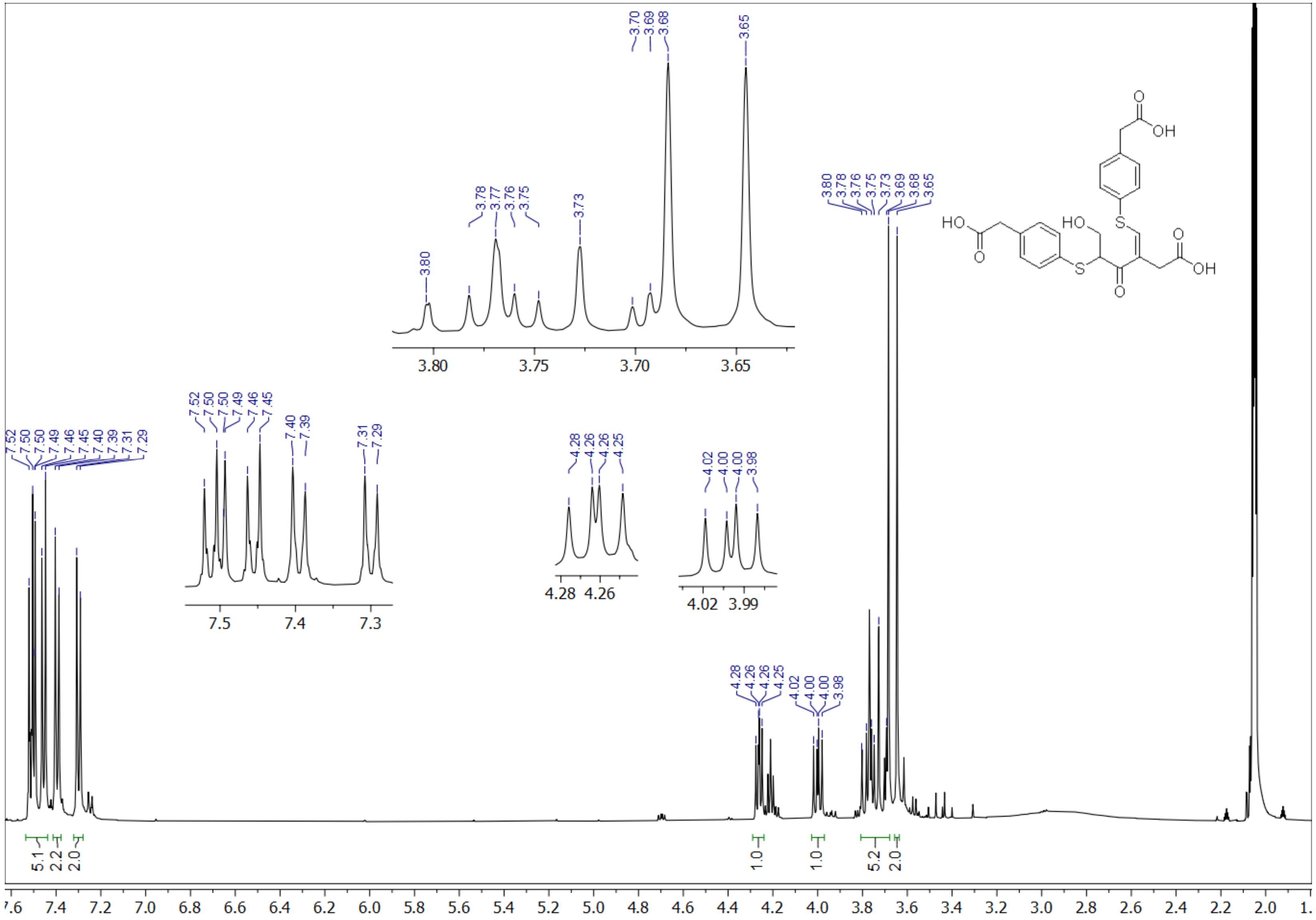

^1H NMR spectrum (300 MHz) of adduct **VI** in acetone d_6

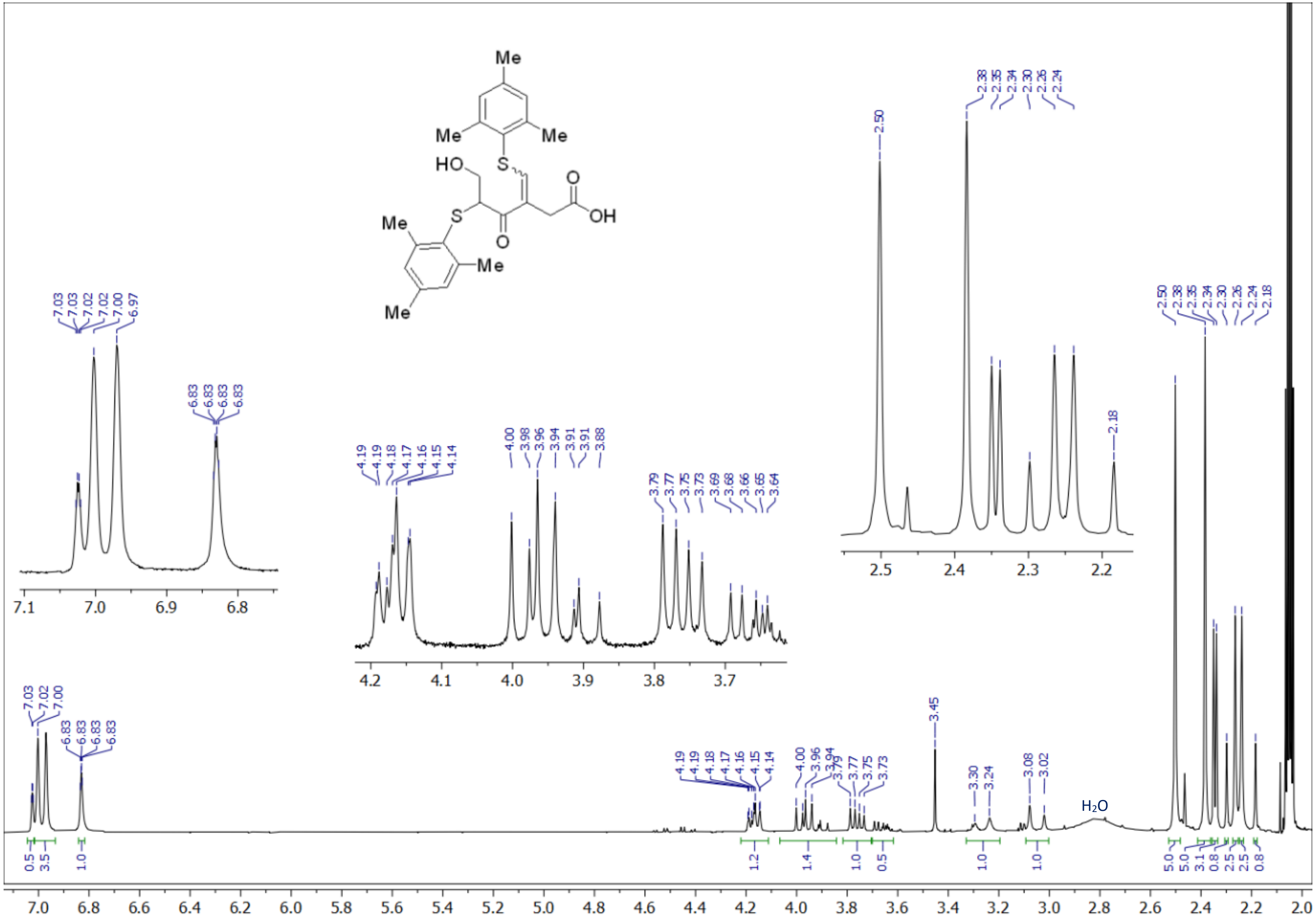

Supplement: Supplementary file 1 — Supplementary Information. [file 41598_2021_2916_MOESM1_ESM.pdf]
